# Insights into the regulatory role of epigenetics in moyamoya disease: Current advances and future prospectives

**DOI:** 10.1016/j.omtn.2024.102281

**Published:** 2024-07-19

**Authors:** Shuangxiang Xu, Tongyu Chen, Jin Yu, Lei Wan, Jianjian Zhang, Jincao Chen, Wei Wei, Xiang Li

**Affiliations:** 1 Brain Research Center, Zhongnan Hospital of Wuhan University, Wuhan University, Wuhan 430071, China; 2 Department of Neurosurgery, Zhongnan Hospital of Wuhan University, Wuhan 430071, China; 3 Frontier Science Center for Immunology and Metabolism, Wuhan University, Wuhan 430071, China; 4 Medical Research Institute, Wuhan University, Wuhan 430071, China; 5 Sino-Italian Ascula Brain Science Joint Laboratory, Wuhan University, Wuhan 430071, China

**Keywords:** MT: Novel therapeutic targets and biomarker development Special Issue, moyamoya disease, epigenetics, DNA methylation, histone modification, non-coding RNA

## Abstract

Moyamoya disease (MMD) is a progressive steno-occlusive cerebrovascular disorder that predominantly affecting East Asian populations. The intricate interplay of distinct and overlapping mechanisms, including genetic associations such as the RNF213-p.R4810K variant, contributes to the steno-occlusive lesions and moyamoya vessels. However, genetic mutations alone do not fully elucidate the occurrence of MMD, suggesting a potential role for epigenetic factors. Accruing evidence has unveiled the regulatory role of epigenetic markers, including DNA methylation, histone modifications, and non-coding RNAs (ncRNAs), in regulating pivotal cellular and molecular processes implicated in the pathogenesis of MMD by modulating endothelial cells and smooth muscle cells. The profile of these epigenetic markers in cerebral vasculatures and circulation has been determined to identify potential diagnostic biomarkers and therapeutic targets. Furthermore, *in vitro* studies have demonstrated the multifaceted effects of modulating specific epigenetic markers on MMD pathogenesis. These findings hold great potential for the discovery of novel therapeutic targets, translational studies, and clinical applications. In this review, we comprehensively summarize the current understanding of epigenetic mechanisms, including DNA methylation, histone modifications, and ncRNAs, in the context of MMD. Furthermore, we discuss the potential challenges and opportunities that lie ahead in this rapidly evolving field.

## Graphical abstract

**Figure:**
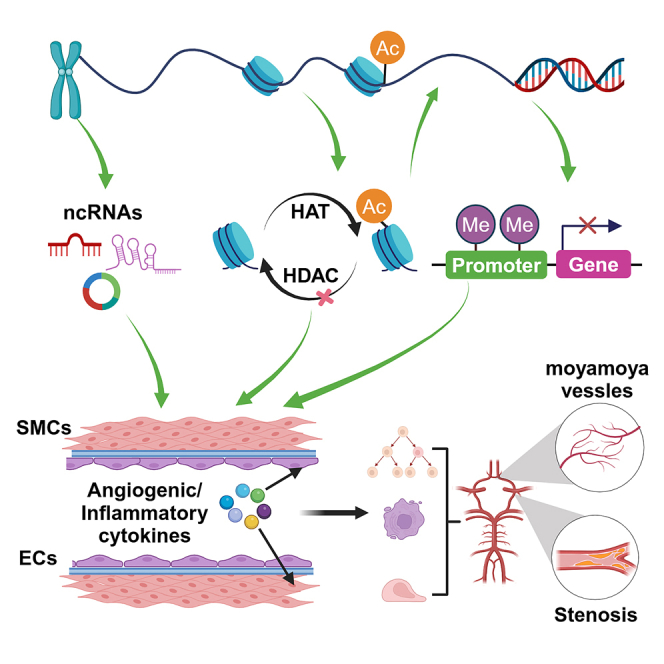

## Teaser

Li and colleagues summarize the latest findings on the epigenetic mechanisms involved in moyamoya disease (MMD), including DNA methylation, histone modifications, and non-coding RNAs (miRNAs, lncRNAs, and circRNAs). These mechanisms dynamically regulate gene expression and play a crucial role in the pathogenesis of MMD, providing valuable insights into its underlying processes.

## Introduction

Moyamoya disease (MMD) poses a significant challenge in the realm of cerebrovascular disorders, characterized by bilateral stenosis of the distal intracranial internal carotid arteries, accompanied by the formation of collateral vessels termed moyamoya vessels.^1^ MMD is widely recognized as one of the most prevalent pediatric cerebrovascular diseases in East Asia, exhibiting a consistent upward trend in its incidence.^2^ The primary clinical manifestations of MMD are transient ischemic attacks and ischemic strokes caused by large artery occlusion, as well as hemorrhagic strokes resulting from the rupture of moyamoya vessels and associated aneurysms.^1^ The pathogenic mechanism of MMD is currently unknown. Some studies have focused on cells related to intimal thickening and abnormal angiogenesis, such as smooth muscle cells (SMCs), endothelial progenitor cells (EPCs), and endothelial cells (ECs). Indeed, previous research has found abnormalities in the quantity and cellular functions of SMCs, EPCs, and ECs derived from patients with MMD.^3^^,^^4^^,^^5^ Despite these observed cellular features and changes, the underlying mechanisms driving these alterations remain unclear.

Considering that MMD is most common in East Asian countries,^6^^,^^7^^,^^8^ but is rarely observed in Europe and the Americas,^1^ it is likely that both genetic and environmental factors contribute to the progression of the disease. While genetic studies have identified certain MMD-specific genes, such as RNF213, ACTA2, GUCY1A3, etc.,^4^ their presence alone cannot fully account for the occurrence of MMD. For instance, the prevalence of the RNF213 R4810K variant is 0.8%–2.0% in the general population of East Asian countries. However, the prevalence of MMD (approximately 1/10,000 population) differs significantly from that of carriers (1/50 in the general population).^9^ This stark contrast underscores the potential contribution of additional factors, like epigenetics, to the development and progression of MMD.

Epigenetics, encompassing DNA methylation, histone modifications, and non-coding RNAs (ncRNAs), plays a critical role in governing gene expression regulation and cellular adaptability to various signals, conditions, and stressors without altering the underlying DNA sequence.^10^ In contrast to the stable genetic content conserved in the genome, epigenetic markers exhibit reversibility through specific inhibitors. Notably, epigenetic mechanisms display dynamic responsiveness to environmental changes, rendering them attractive targets for therapeutic interventions in human diseases.^11^ In recent years, an increasing body of evidence has indicated the pivotal role of various epigenetic mechanisms in the pathogenesis and progression of cerebrovascular diseases, including ischemic stroke, cerebral hemorrhage, and aneurysm formation. These mechanisms primarily entail the regulation of ECs and SMCs, which are precisely the key cells involved in the pathological changes of MMD.^12^^,^^13^^,^^14^

To date, the utilization of genome-wide approaches for profiling the epigenome has progressively expanded the repertoire of pathological epigenetic alterations observed in MMD. In this narrative review, we provide a comprehensive summary of recent investigations elucidating the underlying mechanisms of epigenetics in MMD pathogenesis, including DNA methylation, histone modification and ncRNAs.

## Genetic mutation in MMD

Studies have shown that the incidence of MMD varies among different ethnic groups, suggesting the involvement of genetic risk factors. Genetic linkage analyses have identified five candidate loci associated with MMD.^15^^,^^16^^,^^17^ Among these, the RNF213 gene has been recognized as the strongest susceptibility gene for MMD, particularly in East Asian populations.^18^^,^^19^^,^^20^ The p.R4810K variant of RNF213 is notably associated with the ischemic type of MMD, while other variants such as p.A4399T are linked to the hemorrhagic type.^21^^,^^22^

In addition to RNF213, other genetic variants have been implicated in MMD. Mutations in the ACTA2 gene are linked to various vascular diseases, including MMD.^3^ Genome-wide association studies have identified SNPs in genes like RAPTOR, CARD14, MTHFR, HDAC9, and TCN2 as being associated with MMD in the Chinese population.^23^ These variants may influence MMD risk through mechanisms such as increased serum homocysteine levels, which are risk factors for coronary heart disease and stroke due to their prothrombotic activity and adverse effects on endothelial function and angiogenesis.^23^^,^^24^

Other genetic factors include GUCY1A3 loss-of-function mutations, which impair vascular SMC response to nitric oxide,^25^ and rare variants in the DIAPH1 gene, which affect actin remodeling in vascular cells.^26^ In addition, variants in genes like CHD4, CNOT3, and SETD5, involved in chromatin remodeling, have been identified in European populations.^27^ This suggests that disrupted chromatin remodeling may predispose individuals to MMD by affecting cell differentiation in the arterial wall or adjacent neuronal cells. Recently, rare variants of the ANO1 gene have been identified through exome sequencing of MMD probands and their affected and unaffected family members. These gain-of-function variants lead to increased sensitivity to intracellular calcium, and are associated with developing typical MMD features, aneurysms and stenosis/occlusion in the posterior circulation.^28^

Overall, much effort has been dedicated to elucidating the pathogenesis of MMD. While certain pathologies can be attributed to genetic mutations, genome-wide association studies have made significant strides in identifying disease-causing genes; however, it is important to note that these loci only account for a minute fraction of cases. The global geographical and demographical distribution of MMD, which exhibits a so-called east-west gradient, indeed raises doubts regarding the potential contribution of environmental factors to the disparities in MMD incidence. Interestingly, an organism’s lifelong exposure to its environment can influence gene expression patterns, potentially resulting in phenotypical alterations that could impact the susceptibility to certain diseases, as demonstrated in earlier studies.^29^^,^^30^ These environmentally mediated changes in expression patterns can be explained by an intricate network of epigenetic marks, including DNA methylation, histone modifications, and ncRNAs. Importantly, some of these epigenetic marks are inheritable, while all exert a reversible impact on gene expression without altering DNA sequences. Due to their susceptibility to environmental influences, these epigenetic patterns serve as a crucial link between life experiences and phenotypes, further emphasizing the interplay between genetics and environment in shaping an individual’s health. To explore the alternative possibility that MMD are caused by environmental influences, many scientists are employing epigenome-wide association studies to detect changes in these epigenetic marks that may potentially be associated with MMD. In the following sections, we delve deeper into the role of these epigenetic marks in MMD, discussing the latest research and advancements in the field.

## DNA methylation

### General introduction of DNA methylation

DNA methylation is a covalent modification that occurs exclusively on cytosine nucleotides, predominantly within the context of cytosine-phosphate-guanine dinucleotides (a 5′ cytosine followed by a 3′ guanine).^31^ DNA methylation is an epigenetic hallmark, a phenomenon characterized as “a heritable and stable phenotype resulting from chromosomal changes without alterations in the DNA sequence.”^32^ Despite the shared DNA sequence across all cell types in the body, DNA methylation involves an intricate mechanism that annotates genetic information, resulting in distinct methylation patterns among diverse cell types. The process of DNA methylation consists of three distinct stages: establishment (*de novo* methylation), maintenance, and demethylation. *De novo* methylation is facilitated by DNA methyltransferases 3A (DNMT3A) and DNMT3B, which catalyze the addition of a methyl group to the fifth carbon atom of cytosine nucleotide, resulting in the formation of 5-methylcytosine (5mC).^33^^,^^34^ 5mC is maintained by a set of factors (DNMT1^35^ and UHRF1^36^^,^^37^) that faithfully re-establish methylation patterns during DNA replication. This mechanism underlies both the heritability and clinical applications associated with DNA methylation. As a result, the presence of DNA methylation on gene regulatory regions, such as promoters and enhancers, leads to transcriptional repression.^38^ This repression is achieved through the inhibition of transcription factor binding or the formation of a compact chromatin structure, thereby rendering the gene less accessible to the transcription machinery.^39^ However, DNA methylation on the gene body tends to facilitate transcription elongation rather than gene silencing, as demonstrated by its common presence in ubiquitously expressed genes and its positive correlation with transcription.^31^ In the active demethylation process, TET proteins can iteratively oxidize 5mC to generate 5-hydroxymethylcytosine (5hmC),^40^^,^^41^ 5-formylcytosine (5fC), and 5-carboxylcytosine (5caC),^42^^,^^43^ while thymine DNA glycosylase is responsible for removing 5fC and 5caC.^42^^,^^44^ Then, through the base excision repair pathway, the 5mC is reversed to its unmodified state.^45^ However, in the absence of functional DNA methylation maintenance mechanisms, 5mC will gradually dilute during DNA replication, a process called passive DNA demethylation. DNA methylation is fundamental to the regulation of diverse cellular processes, including regulating transcription, embryonic development, genomic imprinting, and X chromosome inactivation.^38^

### DNA methylation in MMD

DNA methylation plays an important role in several human diseases. Aberrant DNA methylation is a defining characteristic shared by virtually all cancer types.^46^ DNA methylation exhibits tissue specificity, a feature extends to cancer. This feature leads to a tumor-specific DNA methylation profile, which serves as the basis for using DNA methylation as a tool for cancer classification and diagnosis.^47^ Beyond the scope of cancer, DNA methylation hold significance as gene sequences alone do not provide information about non-cancerous diseases such as neurodegenerative diseases, inflammation, and cerebrovascular diseases. In cerebrovascular diseases: DNA hypermethylation changes occur early in atherosclerotic plaque formation and are not affected by the degree of lesion.^48^ These methylation features can distinguish ruptured and unruptured plaques. However, after cerebrovascular events, the plaques undergo extensive demethylation, thereby promoting the expression of genes with arterial protective functions.^49^

In the context of MMD, Sung et al. reported a decrease in promoter methylation of sortilin1 (SORT1) and upregulation in the messenger RNA (mRNA) levels of SORT1 in endothelial colony-forming cells (ECFCs) derived from MMD patients compared with healthy controls.^50^ SORT1 overexpression enhanced the expression of vascular endothelial growth factor (VEGF), vascular endothelial growth factor receptor-1, basic fibroblast growth factor (bFGF), and matrix metalloproteinase-9 (MMP9), while it inhibited the expression of angiopoietin-1 (Ang-1) and thrombospondin 2 in ECs.^50^ These angiogenic factors collectively contribute to the pathogenesis of MMD by modulating the function of ECs and SMCs. Moreover, Toth et al. demonstrated that the absence of SORT1 in brain ECs resulted in impaired blood-brain barrier through promotion of the mitogen-activated protein kinase (MAPK)/ERK signaling pathway activation.^51^

In addition to its role in regulating angiogenic factors, SORT1 has been implicated in the modulation of proinflammatory cytokine secretion during various immune functions, including cell cytotoxicity and inflammation, by exerting control over the exocytosis of interferon gamma (IFN-γ), IFN-α, IL-6, IL-10, IL-17A, and IL-12.^52^ Recently, another study found that the concentration of SORT1 in serum and cerebrospinal fluid (CSF) was significantly higher in MMD patients than in controls. In addition, in patients with MMD, serum SORT1 exhibited a positive correlation with proinflammatory cytokines such as C-reactive protein, IL-6, and INF-γ, suggesting that SORT1 could serve as a clinically valuable biomarker alongside levels of proinflammatory cytokines.^53^

A recent epigenome-wide association study has demonstrated that patients with ischemic MMD exhibit higher genome-wide methylation levels in their whole blood compared with healthy individuals. KCNMA1 and GALNT2 exhibited increased expression levels among the genes with significant differential expression, whereas SOX6 and RBM33 demonstrated decreased expression levels.^54^ In addition, in their subsequent *in vitro* studies using human brain microvascular ECs, the overexpression of KCNMA1 or GALNT2 was found to significantly enhance cell proliferation and tubule formation, while knockdown of SOX6 or RBM33 resulted in a notable reduction in these processes.^54^ KCNMA1 encodes the α subunit of the large-conductance potassium calcium-activated channel, which plays a crucial role in determining the membrane potential of SMCs and consequently influences vascular activities.^55^ Aberrant KCNMA1 expression, regulated by DNA methylation, may contribute to the pathology of MMD through modulation of vascular tone. The studies suggest that DNA methylation plays a crucial role in pathogenesis by modulating ECs and SMCs' functionality.

## Histone modification

### General introduction of histone modification

A nucleosome is a fundamental building block of chromatin, composed of 147 DNA base pairs that are coiled around a core histone protein octamer. The octamer consists of two sets each of H2A, H2B, H3, and H4 histones. Histone proteins undergo various modifications such as methylation, acetylation, phosphorylation, and ubiquitination; among these modifications, methylation and acetylation have been extensively researched.^56^ The methylation of histones predominantly occurs at lysine and arginine residues, which is dynamically regulated by the interplay between histone methyltransferases and demethylases. Well-established lysine methylation sites include H3K4, H3K9, H3K27, H3K36, H3K79, and H4K20, while arginine methylation sites are comprised of H3R2, H3R8, H3R17, H3R26, and H4R315. The transcriptional impact of histone methylation is site specific; for instance, the active regions are marked by methylation at sites such as H3K4, H3K36, H3K79, and H3R17, whereas repressed regions exhibit methylation at sites such as H3K9, H3K27, and H4K20.^57^^,^^58^

The process of histone acetylation typically involves the addition of an acetyl group to a lysine residue, catalyzed by histone acetyltransferases, or its removal by histone deacetylases (HDACs). Commonly observed sites for lysine acetylation include H3K9, H3K14, H3K27, H4K8, and H4K12. In general, histone acetylation attenuates the interaction between histones and DNA through neutralization of the lysine residue, leading to chromatin structure opening and subsequent transcriptional activation.^57^^,^^58^ Recently, an increasing body of evidence has demonstrated the important role of histone acetylation in the pathogenesis of cerebrovascular diseases.^59^^,^^60^^,^^61^

### Histone acetylation in MMD

In a previous study conducted by Kim et al., a significant reduction in the expression of retinaldehyde dehydrogenase 2 (RALDH2), an enzyme responsible for retinoic acid (RA) metabolism, was observed in ECFCs derived from patients with MMD compared with those from healthy controls.^62^ In addition, knockdown of RALDH2 in normal ECFCs or human umbilical vein endothelial cells significantly attenuated cell capillary formation. Furthermore, they demonstrated that the impaired acetyl-histone H3K27 binding to the promoter region of RALDH2 may account for the reduced expression of RALDH2 in ECFCs from patients with MMD, as demonstrated by chromatin immunoprecipitation assay.^62^ In the subsequent study, they demonstrated that modulation of RALDH2 acetylation by panobinostat, an HDAC inhibitor, effectively restored the impaired angiogenic potential of ECFCs derived from MMD patients.^63^ Bonney et al. demonstrated that RA may exert a distinct, cell-autonomous function in brain ECs, inhibiting transcriptional activity of Wnt-β-catenin and its downstream target SOX17. These findings suggest that RA may function as a regulatory mechanism to impede the endothelial Wnt-β-catenin/SOX17 signaling pathway, thereby ensuring the proper development of brain vasculature.^64^ Therefore, we propose that defective histone acetylation-mediated downregulation of RALDH2 may contribute to aberrant moyamoya vessel formation by reducing RA synthesis and subsequently activating the Wnt-β-catenin/SOX17 signaling pathway in MMD.

In addition, Pinard et al. identified four variants in three genes not previously associated with MMD in the European population: *CHD4*, *CNOT3*, and *SETD5*.^27^ Interestingly, all of these genes were associated with alterations of chromatin structure. *CHD4* encodes a helicase that serves as the primary constituent of nucleosome remodeling and histone deacetylase repressor complex, which couples ATP-dependent chromatin remodeling with histone deacetylase activity, thereby regulating gene expression during brain development.^65^ *CNOT3* is responsible for encoding subunit 3 of the CCR4-NOT transcription complex, which governs the acetylation level of histones H3 and H4. The reversal of heart defects in heterozygous knockout mice has been associated with inhibition of histone acetylases, suggesting a potential link with epigenetic remodeling mechanisms.^66^ *SETD5* closely interacts with histone deacetylase, as demonstrated by an enhanced histone acetylation at transcription start sites upon SETD5 depletion.^67^ These findings suggest that perturbed chromatin remodeling, mediated by histone acetylation level, may represent a potential molecular pathway predisposing individuals to MMD. Further investigations are warranted to ascertain the precise contribution of these genes in the pathogenesis of MMD.

## ncRNAs

### General introduction of ncRNAs

The vast majority, accounting for approximately 95%, of the human genome encodes for ncRNAs, while only a minute fraction (around 1%–2%) is dedicated to encode RNAs that are translated into proteins.^68^ Biologically, ncRNAs regulate the expression of target genes and organize the genome through diverse mechanisms. They also interact with each other, forming a sophisticated and dynamic network of regulatory RNAs. This shifts the traditional view of RNA as a simple intermediary in protein synthesis to recognizing RNA as a functional entity with important regulatory functions. Deciphering the diverse roles and operational functions of ncRNAs is crucial for determining their clinical significance and harnessing their potential as biomarkers or therapeutic targets. Functional studies have indicated that ncRNAs not only regulate essential biological processes like growth, development, and organ function, but also play an important role in a variety of central nervous system (CNS) diseases, such as ischemic stroke, subarachnoid hemorrhage, and neurodegenerative disorders, by regulating angiogenesis, inflammation, cell proliferation, and apoptosis of various cellular types including ECs and SMCs.^69^^,^^70^^,^^71^ The phenotype of vascular smooth muscle cells (VSMCs) undergoes epigenetic modifications in response to stress. For instance, Farina et al. identified a microRNA (miRNA)-mediated pathway involving miR-128 that regulates the expression of Kruppel-like factor 4 (KLF4) and modulates the phenotypic characteristics of VSMCs under both physiological and pathophysiological conditions.^72^ The knowledge mentioned above is being actively translated into clinical applications, rendering them as promising biomarkers and therapeutic targets. Recently, researchers started paying attention to investigate the important roles of ncRNAs in MMD, mainly focused on miRNAs, long non-coding RNAs (lncRNAs), and circular RNAs (circRNAs) (Table 1).

**Table 1:** Summary of ncRNA expression profiles in moyamoya disease

ncRNAs	Year	Main methods	Participants/samples	Up-/downregulated ncRNAs	Validated ncRNAs	Targets/pathways	Reference
miRNA	2014	microarray RT-qPCR	MMD, HC serum	50/44	miR-106b↑ miR-130a↑ miR-126↑ miR-125a-3p↓	RNF 213, BRCC3, angiogenesis	Dai et al.^73^
	2015	RT-qPCR dual-luciferase assay	MMD, HC serum	1/0	miR-let7c↑	RNF 213	Zhao et al.^74^
	2018	microarray RT-qPCR	MMD, HC plasma, iPSECs	151/36	miR-6722-3p↑ miR-328-3p↑	STAT3, IGF1, PTEN signaling	Uchino et al.^75^
	2019	next-generation sequencing RT-qPCR	MMD with/without RNF 213 mutation, HC plasma, exosomes	34/8	miR-199a-3p↑ miR-7641-3p↑ miR-9-5p↓ miR-100-5p↓ miR-122-5p↓ miR-197-3p↑ miR-139-5p↑	angiogenesis, inflammation	Lee et al.^76^
	2020	microarray RT-qPCR	MMD, HC CSF, exosomes	4/1	miR-3679-5p↑ miR-6165↑ miR-6760-5p↑ miR-574-5p↓	cell adhesion, junction formation	Wang et al.^77^
	2021	microarray RT-qPCR	MMD CSF	75/62	miR-92a-3p↑ miR-486-3p↑ miR-25-3p↑ miR-155-5p↑	angiogenesis, prognostic biomarkers	Wang et al.^78^
	2023	next-generation sequencing RT-qPCR	MMD, HC plasma, exosomes	585/417	miR-1306-5p↓ miR-196b-5p↑ miR-19a-3p↑ miR-22-3p↑ miR-320b↑ miR-34a-5p↑ miR-485-3p↑ miR-487b-3p↑ miR-489-3p↓ miR-501-3p↑	axon guidance, regulation of the actin cytoskeleton and the MAPK signaling pathway	Huang et al.^79^
	2023	next-generation sequencing RT-qPCR	MMD, AS-CVD CSF, exosomes	153/98	miR-421↑ miR-361-5p↑	cytoplasmic stress granule	Ota et al.^80^
lncRNA	2016	microarray RT-qPCR	MMD, HC peripheral blood	1494/2155	ENST00000416385↑ ENST00000454968↑ ENST00000453213↑ ENST00000408564↑ ENST00000565401↓ ENST00000409054↑ NR_027252↑ NR_344580↑ ENST00000409898↑ NR_015395↑ NR_024420↑ NR_033908↑	inflammation, Toll-like signaling, cytokine-cytokine receptor interaction, MAPK signaling	Gao et al.^81^
	2020	microarray	aMMD, HC peripheral neutrophils	1338/1486	none	metabolic processes	Han et al.^82^
	2021	microarray RT-qPCR	MMD, epilepsy STA	3862/2382	MSTRG.13633↓ lnc-MSC-6↓ lnc-MSC-AS1↓	angiogenesis, inflammation, apoptosis	Zhao et al.^83^
	2022	Microarray	MMD, aneurysm, epilepsy MCA	306/2	None	antibacterial humoral response, T cell receptor signaling pathway, positive regulation of cytokine production, branching involved in blood vessel morphogenesis	Mamiya et al.^84^
circRNA	2017	microarray RT-qPCR	MMD, HC peripheral blood	29/117	hsa_circRNA_100914↓ hsa_circRNA_103343↓ hsa_circRNA_050898↓ hsa_circRNA_101720↑ hsa_circRNA_067209↑	inflammation, angiogenesis, metabolism, MAPK signaling	Zhao et al.^85^
	2019	microarray RT-qPCR	aMMD, HC peripheral neutrophils	54/69	hsa_circRNA_100146↑ hsa_circRNA_102534↓ hsa_circRNA_036592↑ hsa_circRNA_405463↑ hsa_circRNA_405324↓	inflammation, angiogenesis, metabolism, HIF-1α, MAPK, EGFR, VEGF signaling	Ma et al.^86^
	2023	microarray RT-qPCR	MMD, AS-CVD, HC exosomes	none	hsa_circRNA_0000583↓ hsa_circRNA_0090577↓ hsa_circRNA_0051937↓ hsa_circRNA_0076043↓ hsa_circRNA_0074463↓ hsa_circRNA_0001491↑	immune dysfunction, NOD-like receptor signaling pathway, and Toll-like receptor signaling pathway	He et al.^87^

↑ indicates upregulated; ↓ indicates downregulated; RT-qPCR, reverse transcription quantitative polymerase chain reaction; MMD, moyamoya disease; aMMD, asymptomatic MMD; HC, healthy control; AS-CVD, arteriosclerotic cerebrovascular disease; CSF, cerebrospinal fluid; iPSECs, induced pluripotent stem endothelia cells; STA, superficial temporal artery; MCA, middle cerebral artery.

## miRNAs

### Biogenesis of miRNAs

**Figure 1:**
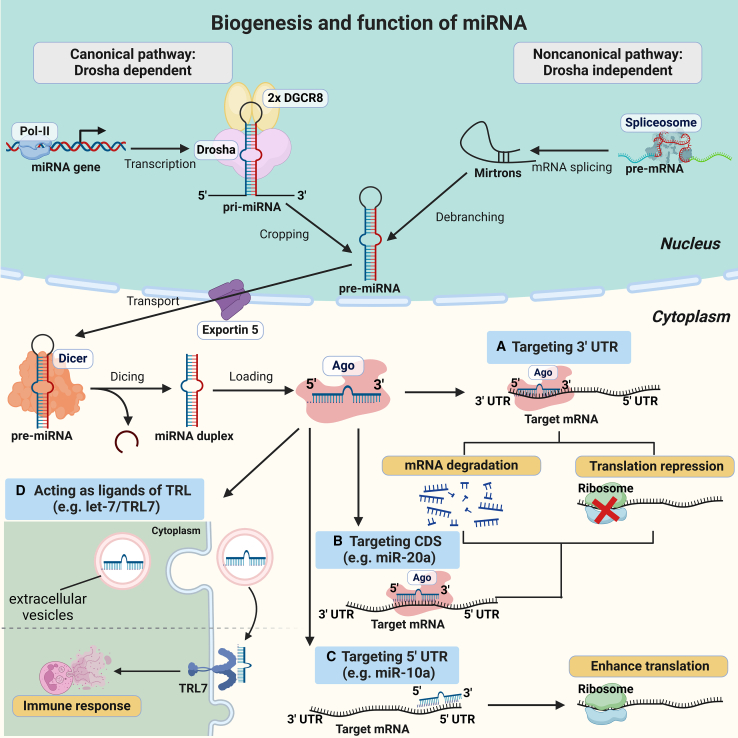
The biogenesis and functions of miRNAs Canonically, miRNA genes are first transcribed by RNA polymerase II (Pol II) in the nucleus, resulting in primary miRNA (pri-miRNAs) transcripts that contain stem-loop structures. Next, the nuclear microprocessor, consisting of RNase III enzyme Drosha and its cofactor DGCR8, identifies and cleaves these stem-loop structures to release a precursor miRNA (pre-miRNA). The pre-miRNA is then transported into the cytoplasm by Exportin-5 and cleaved near the terminal loop by the RNase III enzyme Dicer, leaving an miRNA duplex. The guide strand is loaded into an Argonaute (Ago) protein to form the core of the silencing complex, while the complementary strand is discarded and degraded. Noncanonical processing pathways of miRNA biogenesis involve the generation of pre-miRNA hairpins independent of Drosha through the processing of mirtrons, which are pre-miRNA mimics generated by the spliceosome and intron-debranching enzymes. (A) The canonical function of miRNAs is to target the 3′ untranslated region (UTR) of target mRNAs, causing mRNA degradation or translational repression. (B) Noncanonically, certain miRNAs can target the coding sequence (CDS) of their target mRNA, resulting in mRNA degradation or translational repression, as illustrated for miR-20a binding to the CDS of DAPK3 mRNA causing its translational repression by inducing transient ribosome stalling. (C) A class of miRNAs can also target the 5′ UTR of their target mRNA, resulting in translational activation. For example, miR-10a can interact with the 5′ UTR of mRNAs encoding ribosomal proteins to enhance their translation. (D) Some miRNAs can be secreted into extracellular vesicles and directly target Toll-like receptors (TLRs) by acting as their ligands, in turn activating TLR signaling pathways and inducing an immune response.

miRNAs, a diverse family of short ncRNA typically consisting of 20–24 nucleotides in length, are transcribed by RNA polymerase II (Pol II) as part of longer hairpin-containing RNAs known as primary miRNA (pri-miRNA).^88^ Most canonical miRNAs derive from the introns or exons of non-coding transcripts, some of which harbor hairpins for more than one miRNA. In addition, few miRNAs derive from exons of protein-coding genes.^89^ In mammals, most miRNAs are evolutionarily conserved and are produced through a canonical processing pathway. This pathway involves cleavage of pri-miRNA transcripts by the nuclear microprocessor, a protein complex containing one RNase III enzyme Drosha bound to two copies of its cofactor DGCR8, releasing a precursor miRNA (pre-miRNA) hairpin ∼55–70 nucleotides in length. The pre-miRNA is then transported to the cytoplasm by Exportin 5 and cleaved near the terminal loop by the RNase III enzyme Dicer, leaving an miRNA duplex with two ∼22 nucleotide strands. One strand of each miRNA duplex can be loaded into an Argonaute (Ago) protein to form the core of the silencing complex, which represses the expression of target mRNA, while the complementary strand is discarded and degraded (Figure 1).^89^^,^^90^

Noncanonical processing pathways of miRNA biogenesis involve the generation of pre-miRNA hairpins independent of Drosha through the processing of mirtrons and other pri-miRNAs.^89^^,^^91^ Mirtrons are pre-miRNA mimics generated by the spliceosome and intron-debranching enzymes rather than by Drosha.^92^ In addition, some miRNA genes produce tailed mirtrons, which resemble classical mirtrons except that they have a tail of flanking RNA at either the 5′ or 3′ ends of the pre-miRNA hairpin, which is removed by non-Drosha nucleases before the pre-miRNA enters the pathway.^92^^,^^93^ Knowledge of such atypical pathways enables the design of synthetic substrates that efficiently bypass both RNase III enzymes to generate functional miRNAs.

### Function of miRNAs

It is estimated that approximately 60% of human proteins are under direct regulation by miRNAs, based on conserved base pairing interactions between miRNAs seed (nucleotides 2–8) and their mRNA targets.^88^ Classically, miRNAs exert post-transcriptional control over target gene expression by predominantly recruiting Ago protein to form an essential miRNA-Ago silencing complex, which binds to the 3′ untranslated region (3′ UTR) of a complementary target mRNA. Subsequently, the target mRNA will be cleaved by the miRNA-Ago silencing complex, thereby leading to its degradation or translational repression. (Figure 1)^89^^,^^90^^,^^94^ In general, miRNAs form complex networks of interactions, as one miRNA can silence hundreds of mRNAs, and one mRNA can be regulated by multiple different miRNAs. Furthermore, entire cellular pathways can be regulated by individual miRNAs or miRNA clusters.^95^^,^^96^ In addition, certain different miRNAs have a potential cooperative repression effect on the same target mRNA by binding to near spaced target sites of the mRNA.^97^

Recently, studies have shown that miRNAs also have the ability to regulate gene expression by binding to the coding sequences (CDSs) or to the 5′ UTR of target mRNA.^98^ For example, Zhang et al. demonstrate that a class of miRNAs (miR-20a, miR-17), the target sites of which are located in CDS of *DAPK3*, requires extensive base pairing at the 3′ ends instead of the 5′ seed. Mechanistically, these miRNAs inhibit gene expression also dependent on Ago but independent on GW182 (glycine-tryptophan protein of 182 kDa) proteins, which is necessary for the canonical gene silencing manner of miRNAs, and represses translation by inducing transient ribosome stalling rather than mRNA destabilization.^99^ Moreover, a previous study reported that miR-10a could interact with the 5′ UTR of mRNAs encoding ribosomal proteins to enhance their translation.^100^ Although miRNAs typically inhibit gene expression, there are instances in which they instead boost translation by binding to the CDSs or the 5′ UTR of mRNAs.

In addition to regulating transcription within the cells in which they are produced, miRNAs can act as intercellular communication molecules through their secretion in extracellular vesicles (EVs). For instance, the secreted miRNA let-7 can directly target Toll-like receptor 7 (TLR7) by acting as its ligand, thereby activating TLR signaling transduction pathways and inducing an immune response in Alzheimer’s disease.^101^

Over the decades, miRNAs have been found to play vital roles in the pathogenesis of multiple CNS diseases, including brain tumors, cerebrovascular disorders, brain injuries, and neurodegenerative diseases.^69^^,^^102^^,^^103^

### miRNAs in MMD

Dai et al. demonstrated that the levels of certain serum miRNAs were disrupted in patients with MMD, with an increased level in miR-106b, miR-130a, and miR-126, and a decreased level in miR-125a-3p. These dysregulated miRNAs were associated with concurrent posttranscriptional suppression of RNF213 and BRCC3 protein expression, resulting in impaired angiogenesis and MMD pathogenesis.^73^ Interestingly, miR-126 exerts its pro-angiogenic effects by downregulating the expression of its target, SPRED1, while concurrently modulating the expression of key genes involved in angiogenesis pathways including VEGF, MMP9, Ang-1, and Ang-2.^104^^,^^105^ Furthermore, the inflammatory responses exhibited by ECs undergo dynamic regulation in the context of hypoxia-acidosis is regulated through the suppression of miR-126 expression and conversely enhanced expression of HMGB1 and downstream inflammation markers such as ROS, NADPH oxidase, and TNF-α.^106^ Hartmann et al. demonstrated that miR-126 targeted SPRED1 contributing to GATA2-mediated formation of normal vascular structures. In contrast to GATA2 deficiency, the addition of miR-126 resulted in the normalization of vascular function and expression patterns of TIMP-1 and bFGF, thereby contributing to paracrine effects that promote angiogenesis.^107^ Concurrently, Chen et al. reported that administration of miR-126-5p in the temporal muscle could improve cerebral blood perfusion and the recovery of cognitive function in a chronically ischemic brain rat model, which was treated by encephalo-myo-synangiosis, a common surgical revascularization for MMD. Functionally, overexpression of miR-126-5p promoted EC proliferation and angiogenesis through activating the PI3K/Akt pathway.^108^ These studies indicate that miR-126 may be a promising therapeutic target for MMD.

Zhao et al. reported a significant elevation level in the serum concentration of miRNA let-7c in MMD patients, which exhibited binding affinity toward RNF213. This finding suggests that let-7c may play a role in the MMD pathogenesis by targeting RNF213 and could potentially serve as a diagnostic biomarker for MMD.^74^ In addition, a recent study demonstrated that, under hypoxic conditions, ECs released let-7c, which could activate TLR7 on SMCs, ultimately resulting in the phenotype transformation and vascular wall remodeling in MMD.^109^

Similarly, miR-6722-3p and miR-328-3p, which modulate the STAT3, insulin-like growth factor 1, and phosphatase and tensin homolog signaling pathways in ECs, exhibited aberrant upregulation in a discordant monozygotic twin cohort with MMD.^75^ The involvement of EV miRNAs, such as miR-9-5p, miR-100-5p, and miR-122-5p, may contribute to the pathogenesis of MMD by modulating angiogenic signaling pathways and immune responses. Moreover, dysregulated levels of miR-574-3p in EV-depleted plasma were found to be linked with RNF213 mutations.^76^

Recently, Wang et al. validated four miRNAs (miR-6165, miR-574-5p, miR-3679-5p, miR-6760-5p) derived from the CSF exosome as diagnostic biomarkers for MMD.^77^ They also used a panel of four miRNAs (miR-92a-3p, miR-486-3p, miR-25-3p, and miR-155-5p) in CSF to predict favorable collateral formation after indirect bypass surgery, suggesting miRNAs may serve as potential prognostic biomarkers for MMD patients.^78^ In a similar vein, Ota et al. identified four highly variable miRNAs (miR-421, miR-361-5p, miR-320a, and miR-29b-3p) within the differentially expressed EV-derived miRNAs specific to MMD in CSF. These miRNAs were associated with vascular lesions and processes like cytoplasmic stress granules in the context of MMD.^80^

Most recently, Huang et al. identified 1,002 differentially expressed miRNAs specific to MMD in the plasma exosome, of which 10 miRNAs (miR-1306-5p, miR-196b-5p, miR-19a-3p, miR-22-3p, miR-320b, miR-34a-5p, miR-485-3p, miR-489-3p, miR-501-3p, and miR-487-3p) were found to be associated with the most sensitive and specific diagnostic values for MMD.^79^ Liu et al. found that the expression of miR-151a-3p and miR-125b-5p were significantly upregulated in plasma exosomes derived from MMD patients. Mechanistically, overexpression of miR-151a-3p and miR-125b-5p enhanced cellular proliferation and migration of ECs and induced endothelial-mesenchymal transition *in vivo*.^110^

Regulating the function of ECs and SMCs, as well as angiogenesis, the specific miRNA aberrations in MMD patients not only represent potential therapeutic targets and diagnostic biomarkers but also provide key insights into a comprehensive understanding of the underlying pathological mechanisms in MMD.

## lncRNAs

### Biogenesis of lncRNAs

lncRNAs are defined as a class of endogenous ncRNAs that are longer than 200 nucleotides and lack appreciable protein translation. Similar to mRNAs, most lncRNAs are transcribed by Pol II from various genomic loci with similar chromatin states to mRNA, resulting lncRNAs frequently consist of multiple exons and are capped by 7-methyl guanosine at their 5′ ends and polyadenylated at their 3′ ends.^111^^,^^112^ In general, lncRNAs can be transcribed in sense or antisense directions from introns or exons of overlapping protein-coding genes. It is worthwhile noting that different DNA elements, such as intergenic regions, promoters, and enhancers, are also transcribed into several distinct classes of lncRNAs in eukaryotic genomes.^111^ However, there are also generally different hallmarks between lncRNAs and mRNAs: lncRNAs usually tend to be shorter, contain fewer but longer exons, are often retained in the nucleus, and their abundance can be several times lower than mRNAs.^113^

Recent studies have paid attention to uncovering different subcellular localizations of lncRNAs, which play pivotal roles on their cellular fates and functions. Many lncRNAs are dominantly localized in the cell nucleus and remain tethered at sites of transcription and chromatin domains, where they are associated with chromatin remodeling, and transcriptional and post-transcriptional modifications, thereby regulating the expression of surrounding genes.^114^^,^^115^ Despite a dominant preferential nuclear localization, many lncRNAs play their function in the cytoplasm rather than in the nucleus.^112^ For example, Guo et al. reported that conserved lncRNAs exhibited distinct subcellular localization in human compared with mouse embryonic stem cells (ESCs), of which a greater proportion of lncRNAs are localized in the cytoplasm in human ESCs. Besides, the relatively high evolutionary plasticity of lncRNAs can support species-specific gene expression programs.^116^ In addition, lncRNAs often contain embedded motifs and secondary structures that can recruit certain nuclear factors, which promote the transportation of lncRNAs to diverse subcellular compartments, such as the mitochondria and exosomes.^112^^,^^117^

### Functions of lncRNAs

**Figure 2:**
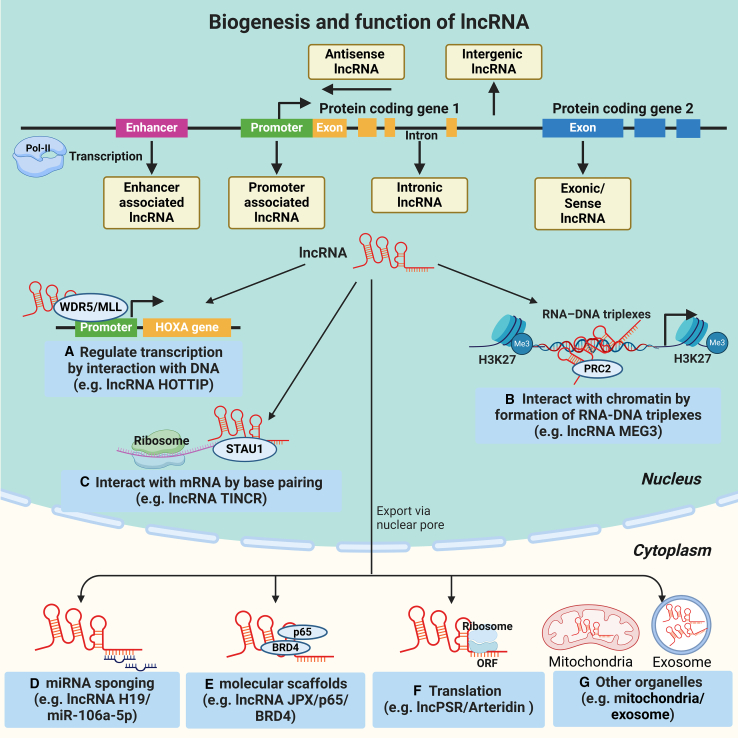
The biogenesis and functions of lcnRNAs Similar to mRNAs, most lncRNAs are transcribed by RNA Pol II from various genomic loci with similar chromatin states to mRNA. In general, lncRNAs can be transcribed in sense or antisense directions from introns or exons of overlapping protein-coding genes. It is worthwhile noting that different DNA elements, such as intergenic regions, promoters, and enhancers, are also transcribed into several distinct classes of lncRNAs in eukaryotic genomes. (A) lncRNAs can modulate chromatin structure and function by interacting with targeted DNA regions. For example, the lncRNA HOTTIP interacts with WD repeat-containing protein 5 (WDR5), thereby guiding the histone methyltransferase complex WDR5--MLL (myeloid/lymphoid leukemia) to the promoters of HOXA genes to promote its transcription. (B) A certain class of lncRNAs play its regulatory activity through the formation of RNA-DNA triplexes. For instance, lncRNA MEG3 possessed GA-rich sequences in binding sites, which guide MEG3 to the chromatin through RNA-DNA triplex formation, and recruit PRC2 to distal regulatory elements, thereby establishing H3K27me3 marks to modulate the activity of TGF-β genes. (C) Some lncRNAs can directly combine other RNAs by base pairing and affect RNA stability. For example, lncRNA TINCR contains several 25-nucleotide motifs that base pair with complementary sequences in differentiation mRNAs and then recruit STAU1 (Staufen homolog 1) to group TINCR-STAU1 complex to stabilize the differentiation mRNAs. (D) Acting as a miRNA sponge, as illustrated for lncRNA H19 competitively sponging miR-106a-5p to activate Runx2 (runt-related transcription factor-2)-dependent VSMC osteogenic differentiation and vascular calcification. (E) Certain lncRNAs can serve as molecular scaffolds to provide a platform for protein interactions. For example, lncRNA JPX acts as a molecular scaffold for the local recruitment of the chromatin remodeling complex comprising phosphorylated p65 and BRD4 (bromodomain-containing protein 4) to the enhancers of the senescence-associated secretory phenotype (SASP) gene, activating the transcription of SASP and promoting cellular senescence in VSMCs. (F) Some lncRNAs have small open reading frames that can be translated by ribosomes to encode peptides. For example, lncPSR encoded a peptide named Arteridin, which could regulate downstream genes (such as *ACTA2*, *KLF5*) by directly interacting with a transcription factor YBX1 (Y-box binding protein 1) and modulating its nuclear translocation and chromatin targeting, thus inducing VSMC phenotype switching and vascular remodeling. (G) In the cytoplasm, lncRNAs can undergo specific sorting processes that assign different lncRNAs to specific organelles (e.g., mitochondria, exosomes).

lncRNAs have been recognized as important regulators in the modulation of protein-coding gene expression and function through diverse mechanisms, including facilitation of gene expression, protein translocation, and post-transcriptional modification via specific sequence motifs and secondary structures that interact with target DNA, RNA, or proteins (Figure 2).^118^^,^^119^^,^^120^

Since numerous lncRNAs are localized in chromatin, they can modulate chromatin structure and function by regulating certain protein binding and activity at targeted DNA regions. For example, the lncRNA HOTTIP derived from the HOXA locus directly interacts with WD repeat-containing protein 5 (WDR5), thereby guiding the histone methyltransferase complex WDR5-MLL (myeloid/lymphoid leukemia) to the promoters of HOXA genes to promote gene transcription.^121^ Moreover, a certain class of lncRNAs have been proposed to play their regulatory activity through the formation of RNA-DNA triplexes, which are essential for promoting or inhibiting gene expression.^112^ Mondal et al. reported that lncRNA MEG3 possessed GA-rich sequences in binding sites, which guide MEG3 to the chromatin through RNA-DNA triplex formation, and recruit PRC2 to distal regulatory elements, thereby establishing H3K27me3 marks to modulate the activity of transforming growth factor β (TGF-β) genes.^122^

Due to the specific sequence motifs, some lncRNAs can directly combine other RNAs by base pairing and subsequently recruit proteins to form regulatory complexes involved in mRNA degradation. For example, the lncRNA TINCR contains several 25-nucleotide motifs that base pair with complementary sequences in differentiation mRNAs and then recruit Staufen homolog 1 (STAU1) to the group TINCR-STAU1 complex to stabilize the differentiation mRNAs.^123^ Some abundant lncRNAs harboring miRNA binding sites can regulate gene expression as competitive endogenous RNAs to adsorb miRNA, thereby reducing miRNA availability to target mRNAs.^112^^,^^124^ Li et al. revealed that upregulated lncRNA H19 activated Runx2 (runt-related transcription factor-2)-dependent VSMC osteogenic differentiation and vascular calcification through competitively sponging miR-106a-5p.^125^

In addition to binding to sequence motifs, several lncRNAs have been proposed to serve as molecular scaffolds to provide a platform for facilitating or blocking interactions among various molecules and proteins to consist of diverse regulatory complexes, which in turn modulate the transcription of neighboring genes. For example, a newly identified lncRNA JPX has been reported to act as a molecular scaffold for the local recruitment of the chromatin remodeling complex comprising phosphorylated p65 and BRD4 (bromodomain-containing protein 4) to the enhancers of the senescence-associated secretory phenotype (SASP) gene, activating the transcription of SASP and promoting cellular senescence in VSMCs.^126^

Surprisingly, with the development of omics technology, some lncRNAs have been proposed to have small open reading frames that can be translated by ribosomes to encode peptides, some of which play important roles in physiological and pathological processes by interacting with their targets to modulate transcriptional or signaling axis.^127^ Yu et al.^128^ demonstrated that the lncPSR transcribed by PSR (phenotype switching regulator) gene encoded a peptide named Arteridin, which could regulate downstream genes (such as *ACTA2*, *KLF5*) by directly interacting with a transcription factor YBX1 (Y-box binding protein 1) and modulating its nuclear translocation and chromatin targeting, thus inducing VSMC phenotype switching and vascular remodeling.

Cumulatively, lncRNAs have demonstrated their involvement in the pathogenesis of various CNS disorders, including glioma, cerebrovascular disorders, and neurodegenerative diseases.^129^^,^^130^

### lncRNAs in MMD

Current studies have identified a plethora of dysregulated lncRNAs in blood samples from patients with MMD, as compared with healthy controls, using microarray analysis. The subsequent bioinformatic analysis has revealed that the identified lncRNAs participated in key molecular signaling pathways associated with MMD, including inflammation, vasculogenesis, smooth muscle contraction, cytokine-cytokine receptor interaction, metabolism, MAPK, and Toll-like signaling.^81^^,^^82^^,^^131^ Zhao et al. identified 6,235 differentially expressed lncRNAs in the superficial temporal artery in MMD patients. Importantly, these dysregulated lncRNAs demonstrated significant associations with vascular remodeling processes in MMD.^83^ Another study identified 308 dysregulated lncRNAs in middle cerebral artery obtained from MMD patients. These dysregulated lncRNAs were associated with diverse biological processes, including T cell receptor signaling pathway, positive regulation of cytokine production, and blood vessel morphogenesis branching.^132^ These findings suggest that aberrant lncRNAs may provide a new theoretical basis for elucidating the pathogenesis of MMD.

The expression of NR_015395, also known as MIR4435-2HG, was upregulated in MMD patients compared with health controls.^81^ An earlier study identified MIR4435-2HG as a miRNA sponge of miR-528 and miR-202 for TGF-β1, thus activating TGF-β signaling to promote the proliferation and migration ability of NSCLC cells.^133^ The expression of TGF-β1 in SMCs derived from MMD patients was significantly increased.^134^ Furthermore, the overexpression of TGF-β1 in SMCs induces an elevation of connective tissue genes, thereby fostering elastin synthesis and promoting angiogenesis in MMD.^135^ In addition, the dysregulated circulating Treg cells have been demonstrated to upregulate the expression of TGF-β1, thereby exerting an influence on the VEGF signaling pathway and contributing to the aberrant angiogenesis observed in MMD.^136^ Moreover, Hartana et al. confirmed the role of MIR4435-2HG for enhancing immunometabolism activities of myeloid dendritic cells through targeting histone acetylation of RAPTOR, the main component of the mammalian target of the rapamycin complex-1 signaling pathway.^137^ Similarly, Duan et al. identified that RAPTOR was associated with MMD.^23^ Conditional deletion of RAPTOR in ECs and SMCs significantly impaired vascular function, such as the relaxation responses evoked by acetylcholine in aorta.^138^ Therefore, whether MIR4435-2HG has a potential role in the pathogenesis of MMD is valuable to be investigated in the future.

Accumulating evidence has demonstrated that dysregulation of the ECM plays a crucial role in the pathogenesis of MMD, primarily mediated by an imbalanced regulation between MMPs and tissue inhibitors of metalloproteinases.^139^^,^^140^ The expression of NR_038366, also known as HOTAIRM1, was downregulated in asymptomatic MMD patients.^82^ It has been proved that HOTAIRM1 competes with miR-30d-3p to recruit YY1 transcription factor, leading to the upregulation of heat shock transcription factor 1 expression and promoting ECM remodeling in hypoxia-induced alveolar epithelial cells.^141^ Despite the lack of direct relevance to MMD, this finding lends support to the hypothesis that lncRNAs may exert influence on vascular stenosis and the development of moyamoya vessels by regulating peri-endothelial ECM. Further investigations are warranted to validate this hypothesis.

Zhao et al. found that the expression of lncRNA MSC-AS1 and mRNA WNT5A was significantly downregulated in MMD patients. Furthermore, overexpressed WNT5A promoted the tube formation and migration of ECs,^83^ indicating that MSC-AS1 may play a potential role in the pathogenesis of MMD by regulating the function of ECs via the WNT5A/β-catenin signaling pathway.

## circRNAs

### Biogenesis of circRNAs

**Figure 3:**
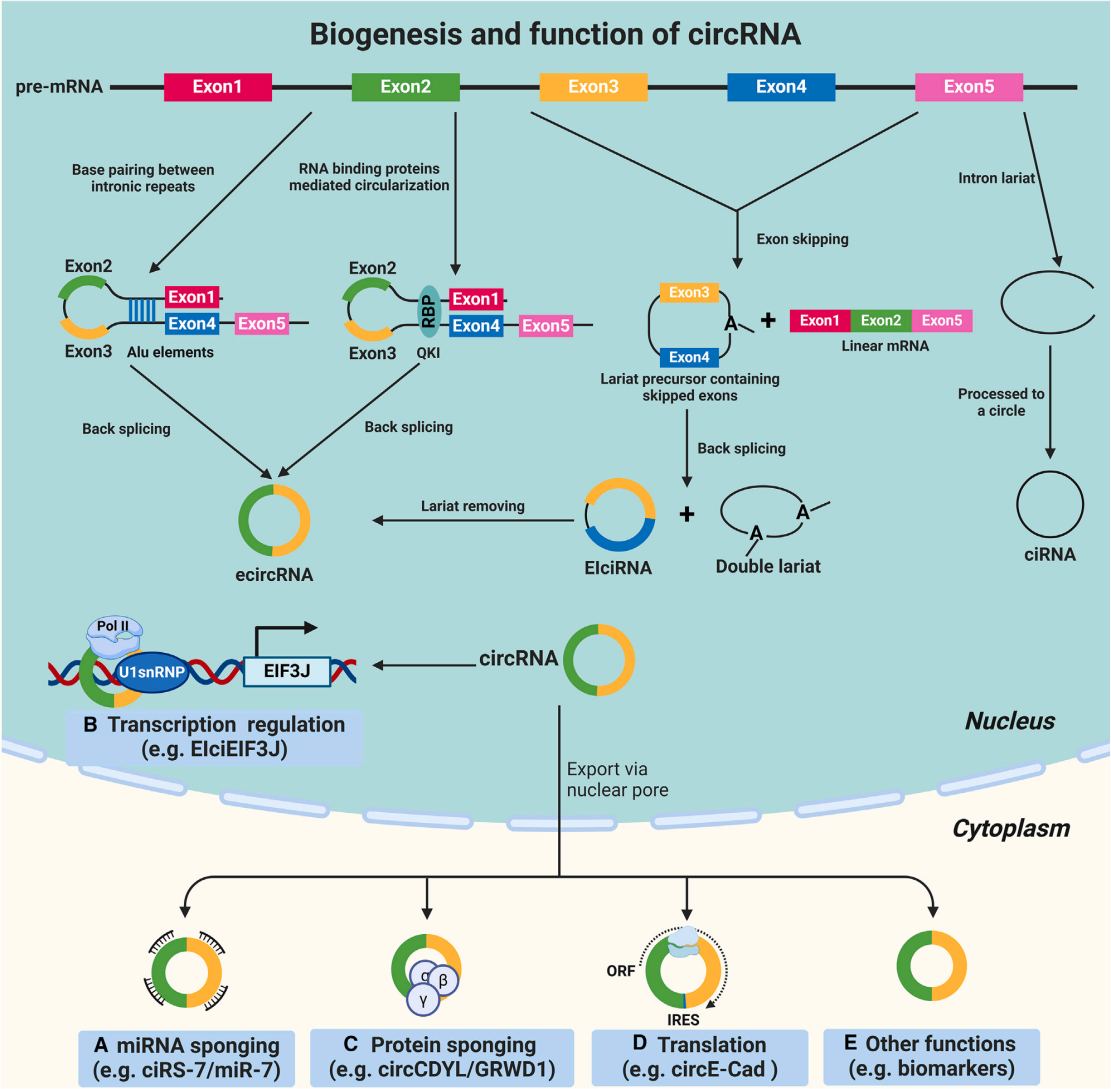
The biogenesis and functions of circRNAs circRNAs are produced by precursor mRNA (pre-mRNA) through a “back-splicing” process, where the downstream 5′ splicing donor is connected to the upstream 3′ splicing acceptor via a 3' → 5′ phosphodiester bond at the back-splicing junction site. The circularization of the exons to form an exonic circRNAs (ecircRNAs) is mediated by the flanking inverted complementary repeats sequence (such as Alu elements) and RNA binding proteins (RBPs). Another formation of circRNAs is associated with exon skipping, in which a lariat precursor containing one or more skipped exons is first generated. Then the lariat removes its internal intron sequences, generating a mature circRNA and a double lariat. In some cases, the intervening introns in the encircled exons are not removed, which produces so-called EIciRNAs. (A) Classically, circRNAs can serve as sponges for miRNAs, as demonstrated by ciRS-7, which harbors target sites for miR-7. (B) A class of nuclear-localized EIciRNAs could regulate the transcription of their parental genes via specific RNA-RNA interaction. For example, EIciEIF3J can control the transcription level of its parent gene EIF3J by interacting with the Pol II and U1 small ribonucleoprotein particle (U1snRNP). (C) Certain circRNAs are also able to act as protein sponges. For example, circCDYL was almost completely covered with binding sites of a multifunctional RBP GRWD1. The interaction between circCDYL and GRWD1 could negatively regulate the key cancer gene *TP53* and promote tumorigenesis. (D) circRNAs can undergo translation and produce small peptides, as demonstrated by circE-Cad encoding a specific secretory E-cadherin protein variant, which contains a unique 14-amino-acid C terminus. (E) Some circRNAs can be secreted in various physiological fluids (including blood, plasma, CSF, and urine samples) and may serve as novel diagnostic and prognostic biomarkers.

circRNAs, a special type of ncRNA characterized by a covalently closed loop structure without 5′ end caps and 3′ poly(A) tails, were first identified in RNA viruses in 1976.^142^ Subsequently, with the advancement and progression of high-throughput RNA sequencing and microarray technologies, a plethora of circRNAs have been discovered in various species in nature.^143^^,^^144^ In general, circRNAs are generated by pre-mRNA through the “back-splicing” process, where the downstream 5′ splicing donor is connected to the upstream 3′ splicing acceptor via a 3' → 5′ phosphodiester bond at the back-splicing junction site.^145^ Generally, circRNAs can be divided into three main categories based on their components: exonic circRNAs (ecircRNAs), intronic circRNAs, and exon-intron circRNAs (EIciRNAs), among which ecircRNAs are the predominant type (Figure 3A).^146^^,^^147^^,^^148^

RNA Pol II elongation rate has been found to correlate with the efficiency and outcome of splicing. The rapid extension of Pol II may promote back-splicing of reverse complementary sequences across long flanking introns, thereby facilitating the formation of circRNAs.^149^ A previous study found that some certain introns have a unique flanking inverted complementary repeats sequence (such as Alu elements), which were necessary for the circularization of the intervening exons. In this process, the Alu elements will be base paired with each other to bring the splicing donor and acceptor close together, thus promoting back-splicing.^150^ Another form of circRNAs generation is associated with exon skipping, in which a lariat precursor containing one or more skipped exons is first generated. Then the lariat removes its internal intron sequences, generating a mature circRNAs and a double lariat.^151^ In some cases, the intervening introns in the encircled exons are not removed, which produces so-called EIciRNAs.^147^ Moreover, the formation of circRNAs sometimes relies on the assistance of RNA binding proteins (RBPs). For instance, the alternative splicing factor Quaking (QKI) can promote back-splicing of pre-mRNA to produce circRNAs by enhancing the interaction between upstream and downstream introns.^152^

### Functions of circRNAs

Initially considered as mere byproducts of RNA processing without any discernible function, circRNAs have emerged as important participants in diverse physiological and pathological processes through their roles as miRNA sponges, transcription regulators, protein sponges, translation templates, and biomarkers (Figure 3B).^145^^,^^148^^,^^153^

Accumulating studies have proven that circRNAs can regulate gene expression by acting as specific miRNA sponges because they harbor multiple miRNA binding sites in circRNAs.^148^^,^^153^ The antisense circular transcript of the *CDR1* gene (CDR1as, also named ciRS-7) was the first reported functional circRNA, which could be worked as an miR-7 sponge in mammalian brain. Because it contains >60 conserved miR-7 binding sites, CDR1as can strongly inhibit miR-7 activity by specifically binding to miR-7, thereby elevating the expression of miR-7 targets IRS2 and EGFR.^143^

Furthermore, in addition to adsorbing miRNAs, studies have shown that a class of nuclear-localized EIciRNAs could regulate the transcription of their parental genes via specific RNA-RNA interaction. For example, EIciEIF3J contains exons and introns that can control the transcription level of its parent gene *EIF3J* by interacting with the Pol II and U1 small ribonucleoprotein particle (U1snRNP).^147^ Recently, a study reported that EIciLIMK1 enhanced the transcription of its host gene *LIMK1* in *cis* to boost neuronal differentiation.^154^

As circRNAs are enriched in the conserved genomic regions, which mainly harbor protein binding sites, it is highly possible that circRNAs are also able to act as protein sponges. It is reported that circRNAs can interact with RBPs to perform biological functions. Okholm et al. demonstrated that circCDYL was almost completely covered with binding sites of a multifunctional RBP GRWD1. The interaction between circCDYL and GRWD1 could negatively regulate the key cancer gene *TP53* and promote tumorigenesis.^155^ Interestingly, a number of circRNAs have been reported to interact with various none-RBP proteins. It has been demonstrated that circFOXO3 could inhibit the function of cyclin-dependent kinase 2 (CDK2) and cyclin-dependent kinase inhibitor 1 (or p21) by formatting a circFOXO3-p21-CDK2 ternary complex, resulting in repression of cell-cycle progression.^156^

In the past years, circRNAs were considered a class of ncRNAs, but recent studies have confirmed that certain circRNAs also show potential coding capabilities.^145^^,^^153^^,^^157^ For example, the circular E-cadherin RNA (circE-Cad) derived from exons 7–10 of the *CDH1* gene owns an internal ribosomal entry site and multiple round open reading frames. Therefore, circE-Cad could encode a specific secretory E-cadherin protein variant that contains a unique 14-amino acid C terminus, thereby promoting the glioma stem cell tumorigenicity.^158^

Given that circRNAs have a special covalently closed loop structure, it was resistant to RNase-R and RNase-H, which are conventionally used to degrade linear RNA. Therefore, most circRNAs remain stable with longer half-life more than 24 h compared with their corresponding linear RNAs, so they can be secreted in various physiological fluids (including blood, plasma, CSF, and urine samples) and may serve as novel biomarkers for early diagnosis and prognosis of diseases.^153^^,^^157^^,^^159^

To date, accumulated evidence has shown that circRNAs play important roles in diverse physiological and pathological processes, such as immune response and disease, cardiovascular disease, and CNS disease.^160^^,^^161^^,^^162^

### circRNAs in MMD

With the development of detection techniques and bioinformatics, an increasing number of circRNAs have been identified in MMD. In 2017, Zhao et al. conducted the pioneering investigation on the expression profile of circRNAs and identified a total of 146 differentially expressed circRNAs in blood samples from MMD patients.^85^ Through bioinformatic analyses, it was revealed that the differentially expressed circRNAs primarily participate in key biological processes such as angiogenesis, immune responses, and metabolism in MMD. Ma et al. also identified 123 dysregulated circRNAs in asymptomatic MMD patients primarily associated with immune responses, angiogenesis, and metabolism, of which, the HIF-1α, MAPK, ErbB, and VEGF signaling pathways were found to be involved in the pathogenesis of MMD.^86^ These studies indicate that the aberrant expression of circRNAs may play potential roles in the pathogenesis of MMD. However, the studies mentioned above remain in the exploration of the expression profile of circRNAs and furthermore lack direct functional investigation in MMD.

In addition, circSATB2 was identified to be differentially expressed in patients with MMD.^85^^,^^86^ A previous study has demonstrated that circSATB2 enhances the proliferation and differentiation of SMCs by acting as an miR-939 sponge to upregulate STIM1 expression.^163^ Recently, Liu et al.^164^ discovered that circZXDC acts as a sponge for miR-125a-3p, thereby upregulating ABCC6 expression. This regulatory mechanism plays a crucial role in the transdifferentiation of SMCs from a contractive phenotype to a synthetic phenotype, ultimately contributing to the intima thickness observed in MMD.

Recently, He et al. investigated the RNA profiles of exosomes in MMD patients. They also detected differential expression of six circRNAs among significantly differentially expressed RNAs using qPCR. Among these, hsa_circRNA_0000583, hsa_circRNA_0051937 (circPRMT1), and hsa_circRNA_0090577 (circCACNA1F) were downregulated. Furthermore, knockdown of hsa_circRNA_0000583, circPRMT1, and circCACNA1F significantly promoted apoptosis and inhibited the proliferation and tube formation of ECs *in vitro*. These results indicated that the downregulation of hsa_circRNA_0000583, circPRMT1, and circCACNA1F may be related to vascular occlusion in MMD.^87^ These findings suggest that investigating aberrant circRNAs in MMD holds a promise as a valuable approach for comprehending the progression of MMD.

## Discussion and perspectives

The above findings demonstrate the wealth of studies investigating the profiles and roles of epigenetic markers pertinent to MMD. Epigenetic mechanisms play a crucial role in gene regulation. Both global and gene-specific DNA methylation contribute to defining distinct epigenetic patterns that have been extensively studied in relation to gene regulation. In addition to DNA methylation, there are other intriguing epigenetic mechanisms like post-translational histone modifications and RNA-mediated processes involving ncRNAs that regulate gene expression. The aberrant proliferation, apoptosis, and migration of ECs and SMCs mediated by epigenetics may be recognized as an important mechanism contributing to the intracranial vessel stenosis/occlusion and moyamoya vessels formation in MMD. These innovative avenues offer promising opportunities for further exploration into unraveling the underlying causes of MMD while also potentially identifying valuable biomarkers specific to MMD.

Currently, surgical revascularization to restore perfusion and to prevent recurrent stroke either through direct or indirect methods is the mainstay treatment for MMD.^165^^,^^166^^,^^167^ The clinical outcome of the surgery is determined by the development of collateral circulation from extracranial arteries to ischemic brain tissue in the postoperative period.^168^ However, poor postoperative collateral formation (PCF) is observed in up to 36.8% of hemispheres.^169^ Furthermore, PCF is highly imprecise and surgical treatments carry potential risks of perioperative ischemic complications and/or cerebral hyperperfusion syndrome.^170^^,^^171^ Therefore, gaining a more comprehensive understanding of the functional significance of the epigenetics in the pathophysiology of MMD will pave the way for the development of novel biomarkers to achieve preoperative assessment of PCF. This can aid surgeons in making more precise surgical decisions, enhancing patient-centered care, and improving surgical outcomes for MMD patients.

To comprehensively profile the methylome of MMD, only two studies have been conducted so far utilizing Illumina 450K^50^ and 850K^54^ beadchip platforms. However, these methylation arrays cover a limited number of methylation sites, potentially missing crucial methylation sites relevant to MMD. Therefore, future research should aim to map the complete methylome of MMD from a whole-genome perspective. Moreover, these studies on DNA methylation have predominantly focused on 5mC. Recently, increasing evidence has demonstrated that TETs can catalyze the oxidation of 5mC into 5hmC, 5fC, and 5caC.^172^^,^^173^ A previous study suggested that TET3 and 5hmC exert a protective effect against ischemic stroke.^174^ The investigation of TET oxidation derivatives in the pathogenesis of MMD necessitates further exploration, which may facilitate the development of novel treatment strategies targeting these DNA epigenetic modifications.

An emerging and exciting clinical application of DNA methylation lies in the field of diagnostics, where DNA methylation on cell-free DNA (cfDNA) derived from dying cells in blood can provide diagnostic information that is otherwise conventionally obtained through invasive biopsies, thus enabling non-invasive diagnosis.^175^ For instance, cfDNA analysis enables identification of fetal chromosome aberrations in maternal plasma^176^ and detection of specific methylation aberrations in circulating tumor DNA for selection of targeted therapies, or monitoring cancer progression or response to therapy, even when the tumor is inaccessible or with unknown origin.^177^^,^^178^ In addition, DNA from transplanted organs can be detected in the plasma of recipients,^179^ and tracking the presence of donor DNA methylation markers in recipient blood has been used to monitor graft rejection.^180^^,^^181^^,^^182^^,^^183^ The potential association between DNA methylation and MMD suggests the rationality of harnessing cfDNA methylation as a biomarker for MMD. Further research is warranted to explore the feasibility of employing cfDNA methylation as a diagnostic biomarker for MMD.

Currently, our understanding of histone modification in MMD remains limited. Therefore, further efforts are required to investigate the functional role of different histone modifications in MMD. Lately, regulatory approval and clinical implementation have been granted to several HDAC-based diagnostic biomarkers and drugs. For example, the potential of HDACs in treating hematological malignancies and solid tumors is currently under evaluation.^184^^,^^185^ By adopting the treatment paradigms that specifically target histone modifications in various diseases, exploring the potential involvement of histone modification in MMD will ultimately pave the way for innovative therapeutic strategies for MMD aimed at manipulating histone modifications.

Accumulating evidence indicates that ncRNAs play an important role in the pathophysiology of cerebrovascular diseases, including MMD. Notably, the administration of miR-126 may serve as an efficiently adjunctive therapy for indirect revascularization in MMD patients due to its proangiogenic properties.^107^^,^^108^ miRNAs have the potential to serve as promising biomarkers for predicting PCF and aiding the surgeon in selecting an appropriate bypass strategy for MMD.^78^ Therefore, the establishment of a comprehensive network of ncRNAs associated with MMD could complement surgical treatments and serve as prognostic biomarkers for personalized treatment, leading to an enhanced overall outcomes of MMD patients.

However, several obstacles must be addressed before the application of these epigenetic factors in clinical settings. These challenges including genetic variations among patients, the potential degradation of ncRNAs, the impact of diverse environment or internal factors, and limited availability and quality of biomarkers, as well as the time-consuming and cost of routine epigenetic testing in clinical practice. Furthermore, our current understanding of the precise signaling pathway involved in epigenetic regulations that mediate pathogenesis in MMD remains limited due to several inherent limitations. For instance, current advancements in MMD research pertaining to genetics and epigenetics primarily stem from transcriptomic profiling studies utilizing peripheral blood or CSF samples. The inclusion of studies utilizing intracranial artery samples is essential for elucidating the underlying molecular mechanism of MMD. Recently, our research team successfully conducted a transcriptomic analysis using RNA sequencing on intracranial artery samples obtained from MMD patients.^140^ This suggests the potential utility of utilizing intracranial artery samples for epigenetic investigations in MMD. In addition, due to the lack of effective cellular or animal models to directly elucidate the precise molecular mechanisms underlying this disease, further clinical studies employing intracranial artery samples are warranted to explore the possible involvement of epigenetics in MMD. Simultaneously, it is crucial to develop reliable MMD models for facilitating the application of epigenetics-based therapeutics.

The investigation into the impact of epigenetic markers on MMD has made significant advancements in recent years. However, despite these advancements, as demonstrated by the findings discussed in this review, the precise role of each epigenetic marker in MMD remains largely unclear. Therefore, further exploration of diverse facets of MMD epigenetics is imperative from both mechanistic and clinical perspectives, including their potential utilization as diagnostic biomarkers, prognostic indicators, and therapeutic targets.

## References

[1] Kuroda S., Houkin K. (2008). Moyamoya disease: current concepts and future perspectives. Lancet Neurol..

[2] Kim J.S. (2016). Moyamoya Disease: Epidemiology, Clinical Features, and Diagnosis. J. Stroke.

[3] Guo D.C., Papke C.L., Tran-Fadulu V., Regalado E.S., Avidan N., Johnson R.J., Kim D.H., Pannu H., Willing M.C., Sparks E. (2009). Mutations in smooth muscle alpha-actin (ACTA2) cause coronary artery disease, stroke, and Moyamoya disease, along with thoracic aortic disease. Am. J. Hum. Genet..

[4] Chen T., Wei W., Yu J., Xu S., Zhang J., Li X., Chen J. (2023). The Progression of Pathophysiology of Moyamoya Disease. Neurosurgery.

[5] Yu J., Du Q., Hu M., Zhang J., Chen J. (2020). Endothelial Progenitor Cells in Moyamoya Disease: Current Situation and Controversial Issues. Cell Transplant..

[6] Ma Z., Wang X., Li M., Zhou D., Chen J. (2021). An ecological comparison study on the causal association between leptospirosis and moyamoya disease in Hubei, China, 2017-2019. Clin. Neurol. Neurosurg..

[7] Hayashi K., Horie N., Suyama K., Nagata I. (2013). An epidemiological survey of moyamoya disease, unilateral moyamoya disease and quasi-moyamoya disease in Japan. Clin. Neurol. Neurosurg..

[8] Ahn I.M., Park D.H., Hann H.J., Kim K.H., Kim H.J., Ahn H.S. (2014). Incidence, prevalence, and survival of moyamoya disease in Korea: a nationwide, population-based study. Stroke.

[9] Koizumi A., Nagata K., Houkin K., Tominaga T., Miyamoto S., Kure S., Tournier-Lasserve E. (2017).

[10] Katada S., Imhof A., Sassone-Corsi P. (2012). Connecting threads: epigenetics and metabolism. Cell.

[11] Jaenisch R., Bird A. (2003). Epigenetic regulation of gene expression: how the genome integrates intrinsic and environmental signals. Nat. Genet..

[12] Hu Z., Zhong B., Tan J., Chen C., Lei Q., Zeng L. (2017). The Emerging Role of Epigenetics in Cerebral Ischemia. Mol. Neurobiol..

[13] Fang Z., Sun X., Wang X., Ma J., Palaia T., Rana U., Miao B., Ragolia L., Hu W., Miao Q.R. (2022). NOGOB receptor deficiency increases cerebrovascular permeability and hemorrhage via impairing histone acetylation-mediated CCM1/2 expression. J. Clin. Invest..

[14] Fernández-Pérez I., Macias-Gómez A., Suárez-Pérez A., Vallverdú-Prats M., Giralt-Steinhauer E., Bojtos L., Susin-Calle S., Rodriguez-Campello A., Guisado-Alonso D., Jimenez-Balado J. (2024). The Role of Epigenetics in Brain Aneurysm and Subarachnoid Hemorrhage: A Comprehensive Review. Int. J. Mol. Sci..

[15] Bang O.Y., Fujimura M., Kim S.K. (2016). The Pathophysiology of Moyamoya Disease: An Update. J. Stroke.

[16] Yamauchi T., Tada M., Houkin K., Tanaka T., Nakamura Y., Kuroda S., Abe H., Inoue T., Ikezaki K., Matsushima T., Fukui M. (2000). Linkage of familial moyamoya disease (spontaneous occlusion of the circle of Willis) to chromosome 17q25. Stroke.

[17] Sakurai K., Horiuchi Y., Ikeda H., Ikezaki K., Yoshimoto T., Fukui M., Arinami T. (2004). A novel susceptibility locus for moyamoya disease on chromosome 8q23. J. Hum. Genet..

[18] Kamada F., Aoki Y., Narisawa A., Abe Y., Komatsuzaki S., Kikuchi A., Kanno J., Niihori T., Ono M., Ishii N. (2011). A genome-wide association study identifies RNF213 as the first Moyamoya disease gene. J. Hum. Genet..

[19] Liu W., Morito D., Takashima S., Mineharu Y., Kobayashi H., Hitomi T., Hashikata H., Matsuura N., Yamazaki S., Toyoda A. (2011). Identification of RNF213 as a susceptibility gene for moyamoya disease and its possible role in vascular development. PLoS One.

[20] Liu W., Hitomi T., Kobayashi H., Harada K.H., Koizumi A. (2012). Distribution of moyamoya disease susceptibility polymorphism p.R4810K in RNF213 in East and Southeast Asian populations. Neurol. Med.-Chir..

[21] Wu Z., Jiang H., Zhang L., Xu X., Zhang X., Kang Z., Song D., Zhang J., Guan M., Gu Y. (2012). Molecular analysis of RNF213 gene for moyamoya disease in the Chinese Han population. PLoS One.

[22] Guey S., Kraemer M., Hervé D., Ludwig T., Kossorotoff M., Bergametti F., Schwitalla J.C., Choi S., Broseus L., Callebaut I. (2017). Rare RNF213 variants in the C-terminal region encompassing the RING-finger domain are associated with moyamoya angiopathy in Caucasians. Eur. J. Hum. Genet..

[23] Duan L., Wei L., Tian Y., Zhang Z., Hu P., Wei Q., Liu S., Zhang J., Wang Y., Li D. (2018). Novel Susceptibility Loci for Moyamoya Disease Revealed by a Genome-Wide Association Study. Stroke.

[24] Loscalzo J. (2009). Homocysteine-mediated thrombosis and angiostasis in vascular pathobiology. J. Clin. Invest..

[25] Wallace S., Guo D.C., Regalado E., Mellor-Crummey L., Bamshad M., Nickerson D.A., Dauser R., Hanchard N., Marom R., Martin E. (2016). Disrupted nitric oxide signaling due to GUCY1A3 mutations increases risk for moyamoya disease, achalasia and hypertension. Clin. Genet..

[26] Kundishora A.J., Peters S.T., Pinard A., Duran D., Panchagnula S., Barak T., Miyagishima D.F., Dong W., Smith H., Ocken J. (2021). DIAPH1 Variants in Non-East Asian Patients With Sporadic Moyamoya Disease. JAMA Neurol..

[27] Pinard A., Guey S., Guo D., Cecchi A.C., Kharas N., Wallace S., Regalado E.S., Hostetler E.M., Sharrief A.Z., Bergametti F. (2020). The pleiotropy associated with *de novo* variants in CHD4, CNOT3, and SETD5 extends to moyamoya angiopathy. Genet. Med..

[28] Pinard A., Ye W., Fraser S.M., Rosenfeld J.A., Pichurin P., Hickey S.E., Guo D., Cecchi A.C., Boerio M.L., Guey S. (2023). Rare variants in ANO1, encoding a calcium-activated chloride channel, predispose to moyamoya disease. Brain.

[29] Cavalli G., Heard E. (2019). Advances in epigenetics link genetics to the environment and disease. Nature.

[30] Tammen S.A., Friso S., Choi S.W. (2013). Epigenetics: the link between nature and nurture. Mol. Aspect. Med..

[31] Jones P.A. (2012). Functions of DNA methylation: islands, start sites, gene bodies and beyond. Nat. Rev. Genet..

[32] Berger S.L., Kouzarides T., Shiekhattar R., Shilatifard A. (2009). An operational definition of epigenetics. Genes Dev..

[33] Okano M., Xie S., Li E. (1998). Cloning and characterization of a family of novel mammalian DNA (cytosine-5) methyltransferases. Nat. Genet..

[34] Okano M., Bell D.W., Haber D.A., Li E. (1999). DNA methyltransferases Dnmt3a and Dnmt3b are essential for *de novo* methylation and mammalian development. Cell.

[35] Hermann A., Goyal R., Jeltsch A. (2004). The Dnmt1 DNA-(cytosine-C5)-methyltransferase methylates DNA processively with high preference for hemimethylated target sites. J. Biol. Chem..

[36] Bostick M., Kim J.K., Estève P.O., Clark A., Pradhan S., Jacobsen S.E. (2007). UHRF1 plays a role in maintaining DNA methylation in mammalian cells. Science.

[37] Sharif J., Muto M., Takebayashi S.i., Suetake I., Iwamatsu A., Endo T.A., Shinga J., Mizutani-Koseki Y., Toyoda T., Okamura K. (2007). The SRA protein Np95 mediates epigenetic inheritance by recruiting Dnmt1 to methylated DNA. Nature.

[38] Greenberg M.V.C., Bourc'his D. (2019). The diverse roles of DNA methylation in mammalian development and disease. Nat. Rev. Mol. Cell Biol..

[39] Dor Y., Cedar H. (2018). Principles of DNA methylation and their implications for biology and medicine. Lancet.

[40] Tahiliani M., Koh K.P., Shen Y., Pastor W.A., Bandukwala H., Brudno Y., Agarwal S., Iyer L.M., Liu D.R., Aravind L., Rao A. (2009). Conversion of 5-methylcytosine to 5-hydroxymethylcytosine in mammalian DNA by MLL partner TET1. Science.

[41] Ito S., D'Alessio A.C., Taranova O.V., Hong K., Sowers L.C., Zhang Y. (2010). Role of Tet proteins in 5mC to 5hmC conversion, ES-cell self-renewal and inner cell mass specification. Nature.

[42] He Y.F., Li B.Z., Li Z., Liu P., Wang Y., Tang Q., Ding J., Jia Y., Chen Z., Li L. (2011). Tet-mediated formation of 5-carboxylcytosine and its excision by TDG in mammalian DNA. Science.

[43] Ito S., Shen L., Dai Q., Wu S.C., Collins L.B., Swenberg J.A., He C., Zhang Y. (2011). Tet proteins can convert 5-methylcytosine to 5-formylcytosine and 5-carboxylcytosine. Science.

[44] Maiti A., Drohat A.C. (2011). Thymine DNA glycosylase can rapidly excise 5-formylcytosine and 5-carboxylcytosine: potential implications for active demethylation of CpG sites. J. Biol. Chem..

[45] Weber A.R., Krawczyk C., Robertson A.B., Kuśnierczyk A., Vågbø C.B., Schuermann D., Klungland A., Schär P. (2016). Biochemical reconstitution of TET1-TDG-BER-dependent active DNA demethylation reveals a highly coordinated mechanism. Nat. Commun..

[46] Baylin S.B., Jones P.A. (2016). Epigenetic Determinants of Cancer. Cold Spring Harbor Perspect. Biol..

[47] Costello J.F., Frühwald M.C., Smiraglia D.J., Rush L.J., Robertson G.P., Gao X., Wright F.A., Feramisco J.D., Peltomäki P., Lang J.C. (2000). Aberrant CpG-island methylation has non-random and tumour-type-specific patterns. Nat. Genet..

[48] Zaina S., Heyn H., Carmona F.J., Varol N., Sayols S., Condom E., Ramírez-Ruz J., Gomez A., Gonçalves I., Moran S., Esteller M. (2014). DNA methylation map of human atherosclerosis. Circ. Cardiovasc. Genet..

[49] Zaina S., Gonçalves I., Carmona F.J., Gomez A., Heyn H., Mollet I.G., Moran S., Varol N., Esteller M. (2015). DNA methylation dynamics in human carotid plaques after cerebrovascular events. Arterioscler. Thromb. Vasc. Biol..

[50] Sung H.Y., Lee J.Y., Park A.K., Moon Y.J., Jo I., Park E.M., Wang K.C., Phi J.H., Ahn J.H., Kim S.K. (2018). Aberrant Promoter Hypomethylation of Sortilin 1: A Moyamoya Disease Biomarker. J. Stroke.

[51] Toth A.E., Helms H.C., Harazin A., Johnsen K.B., Goldeman C., Burkhart A., Thomsen M.S., Kempen P.J., Klepe A., Lipka D.V. (2022). Sortilin regulates blood-brain barrier integrity. FEBS J..

[52] Talbot H., Saada S., Naves T., Gallet P.F., Fauchais A.L., Jauberteau M.O. (2018). Regulatory Roles of Sortilin and SorLA in Immune-Related Processes. Front. Pharmacol..

[53] Han W., Qiao Y., Zhang H., Geng C., Zhu X., Liao D., Guo Y., Yang M., Chen D., Jiang P. (2021). Circulating sortilin levels are associated with inflammation in patients with moyamoya disease. Metab. Brain Dis..

[54] He S., Ye X., Duan R., Zhao Y., Wei Y., Wang Y., Liu Z., Hao X., Chen X., Hao Q. (2022). Epigenome-Wide Association Study Reveals Differential Methylation Sites and Association of Gene Expression Regulation with Ischemic Moyamoya Disease in Adults. Oxid. Med. Cell. Longev..

[55] Xu T., Zhao M., Li H., Zhou X., Liu B., Sun M., Xu Z., Gao Q. (2020). Antenatal Dexamethasone Exposure Impairs the High-Conductance Ca(2+)-Activated K(+) Channels via Epigenetic Alteration at Gene Promoter in Male Offspring. Arterioscler. Thromb. Vasc. Biol..

[56] Bowman G.D., Poirier M.G. (2015). Post-translational modifications of histones that influence nucleosome dynamics. Chem. Rev..

[57] Kim J., Lee H., Yi S.J., Kim K. (2022). Gene regulation by histone-modifying enzymes under hypoxic conditions: a focus on histone methylation and acetylation. Exp. Mol. Med..

[58] Park J., Lee K., Kim K., Yi S.J. (2022). The role of histone modifications: from neurodevelopment to neurodiseases. Signal Transduct. Targeted Ther..

[59] Su Y., Zhang L., Zhou Y., Ding L., Li L., Wang Z. (2022). The progress of research on histone methylation in ischemic stroke pathogenesis. J. Physiol. Biochem..

[60] Shen Z., Bei Y., Lin H., Wei T., Dai Y., Hu Y., Zhang C., Dai H. (2023). The role of class IIa histone deacetylases in regulating endothelial function. Front. Physiol..

[61] Demyanenko S., Sharifulina S. (2021). The Role of Post-Translational Acetylation and Deacetylation of Signaling Proteins and Transcription Factors after Cerebral Ischemia: Facts and Hypotheses. Int. J. Mol. Sci..

[62] Lee J.Y., Moon Y.J., Lee H.O., Park A.K., Choi S.A., Wang K.C., Han J.W., Joung J.G., Kang H.S., Kim J.E. (2015). Deregulation of Retinaldehyde Dehydrogenase 2 Leads to Defective Angiogenic Function of Endothelial Colony-Forming Cells in Pediatric Moyamoya Disease. Arterioscler. Thromb. Vasc. Biol..

[63] Jangra A., Choi S.A., Koh E.J., Moon Y.J., Wang K.C., Phi J.H., Lee J.Y., Kim S.K. (2019). Panobinostat, a histone deacetylase inhibitor, rescues the angiogenic potential of endothelial colony-forming cells in moyamoya disease. Childs Nerv. Syst..

[64] Bonney S., Harrison-Uy S., Mishra S., MacPherson A.M., Choe Y., Li D., Jaminet S.C., Fruttiger M., Pleasure S.J., Siegenthaler J.A. (2016). Diverse Functions of Retinoic Acid in Brain Vascular Development. J. Neurosci..

[65] Nitarska J., Smith J.G., Sherlock W.T., Hillege M.M.G., Nott A., Barshop W.D., Vashisht A.A., Wohlschlegel J.A., Mitter R., Riccio A. (2016). A Functional Switch of NuRD Chromatin Remodeling Complex Subunits Regulates Mouse Cortical Development. Cell Rep..

[66] Neely G.G., Kuba K., Cammarato A., Isobe K., Amann S., Zhang L., Murata M., Elmén L., Gupta V., Arora S. (2010). A global *in vivo* Drosophila RNAi screen identifies NOT3 as a conserved regulator of heart function. Cell.

[67] Osipovich A.B., Gangula R., Vianna P.G., Magnuson M.A. (2016). Setd5 is essential for mammalian development and the co-transcriptional regulation of histone acetylation. Development.

[68] (2012). An integrated encyclopedia of DNA elements in the human genome. Nature.

[69] Mahjoubin-Tehran M., Rezaei S., Jesmani A., Birang N., Morshedi K., Khanbabaei H., Khan H., Piranviseh A., Nejati M., Aschner M., Mirzaei H. (2021). New epigenetic players in stroke pathogenesis: From non-coding RNAs to exosomal non-coding RNAs. Biomed. Pharmacother..

[70] Zhang L., Li Z., Mao L., Wang H. (2022). Circular RNA in Acute Central Nervous System Injuries: A New Target for Therapeutic Intervention. Front. Mol. Neurosci..

[71] Yang R., Yang B., Liu W., Tan C., Chen H., Wang X. (2023). Emerging role of non-coding RNAs in neuroinflammation mediated by microglia and astrocytes. J. Neuroinflammation.

[72] Farina F.M., Hall I.F., Serio S., Zani S., Climent M., Salvarani N., Carullo P., Civilini E., Condorelli G., Elia L., Quintavalle M. (2020). miR-128-3p Is a Novel Regulator of Vascular Smooth Muscle Cell Phenotypic Switch and Vascular Diseases. Circ. Res..

[73] Dai D., Lu Q., Huang Q., Yang P., Hong B., Xu Y., Zhao W., Liu J., Li Q. (2014). Serum miRNA signature in Moyamoya disease. PLoS One.

[74] Zhao S., Gong Z., Zhang J., Xu X., Liu P., Guan W., Jing L., Peng T., Teng J., Jia Y. (2015). Elevated Serum MicroRNA Let-7c in Moyamoya Disease. J. Stroke Cerebrovasc. Dis..

[75] Uchino H., Ito M., Kazumata K., Hama Y., Hamauchi S., Terasaka S., Sasaki H., Houkin K. (2018). Circulating miRNome profiling in Moyamoya disease-discordant monozygotic twins and endothelial microRNA expression analysis using iPS cell line. BMC Med. Genom..

[76] Lee M.J., Fallen S., Zhou Y., Baxter D., Scherler K., Kuo M.F., Wang K. (2019). The Impact of Moyamoya Disease and RNF213 Mutations on the Spectrum of Plasma Protein and MicroRNA. J. Clin. Med..

[77] Wang G., Wen Y., Faleti O.D., Zhao Q., Liu J., Zhang G., Li M., Qi S., Feng W., Lyu X. (2020). A Panel of Exosome-Derived miRNAs of Cerebrospinal Fluid for the Diagnosis of Moyamoya Disease. Front. Neurosci..

[78] Wang G., Wen Y., Chen S., Zhang G., Li M., Zhang S., Qi S., Feng W. (2021). Use of a panel of four microRNAs in CSF as a predicted biomarker for postoperative neoangiogenesis in moyamoya disease. CNS Neurosci. Ther..

[79] Huang D., Qi H., Yang H., Chen M. (2023). Plasma exosomal microRNAs are non-invasive biomarkers of moyamoya disease: A pilot study. Clinics.

[80] Ota S., Yokoyama K., Kanamori F., Mamiya T., Uda K., Araki Y., Wakabayashi T., Yoshikawa K., Saito R. (2023). Moyamoya disease-specific extracellular vesicle-derived microRNAs in the cerebrospinal fluid revealed by comprehensive expression analysis through microRNA sequencing. Acta Neurochir..

[81] Gao F., Yu L., Zhang D., Zhang Y., Wang R., Zhao J. (2016). Long Noncoding RNAs and Their Regulatory Network: Potential Therapeutic Targets for Adult Moyamoya Disease. World Neurosurg..

[82] Han Z., Li L., Liu P., Huang Y., Zhang S., Li G., Li F., Zhao H., Tao Z., Wang R. (2020). Metabolic Adjustments by LncRNAs in Peripheral Neutrophils Partly Account for the Complete Compensation of Asymptomatic MMD Patients. CNS Neurol. Disord.: Drug Targets.

[83] Zhao J., Qiu C., Zhang G., Chen L., He S., Ma J. (2021). LncRNA-mRNA Co-expression Profiles Relative to Vascular Remodeling in Moyamoya Patients Without RNF213 Mutation. World Neurosurg..

[84] Mamiya T., Kanamori F., Yokoyama K., Ota A., Karnan S., Uda K., Araki Y., Maesawa S., Yoshikawa K., Saito R. (2023). Long noncoding RNA profile of the intracranial artery in patients with moyamoya disease. J. Neurosurg..

[85] Zhao M., Gao F., Zhang D., Wang S., Zhang Y., Wang R., Zhao J. (2017). Altered expression of circular RNAs in Moyamoya disease. J. Neurol. Sci..

[86] Ma Q., Li L., Yu B., Jiao L., Han Z., Zhao H., Li G., Ma Y., Luo Y. (2019). Circular RNA profiling of neutrophil transcriptome provides insights into asymptomatic Moyamoya disease. Brain Res..

[87] He S., Liang J., Xue G., Wang Y., Zhao Y., Liu Z., Hao X., Wei Y., Chen X., Wang H. (2023). RNA profiling of sEV (small extracellular vesicles)/exosomes reveals biomarkers and vascular endothelial dysplasia with moyamoya disease. J. Cerebr. Blood Flow Metabol..

[88] Ebert M.S., Sharp P.A. (2012). Roles for microRNAs in conferring robustness to biological processes. Cell.

[89] Bartel D.P. (2018). Metazoan MicroRNAs. Cell.

[90] Czech B., Hannon G.J. (2011). Small RNA sorting: matchmaking for Argonautes. Nat. Rev. Genet..

[91] Shang R., Lee S., Senavirathne G., Lai E.C. (2023). microRNAs in action: biogenesis, function and regulation. Nat. Rev. Genet..

[92] Ruby J.G., Jan C.H., Bartel D.P. (2007). Intronic microRNA precursors that bypass Drosha processing. Nature.

[93] Khanal S., de Cruz M., Strickland B., Mansfield K., Lai E.C., Flynt A. (2024). A tailed mirtron promotes longevity in Drosophila. Nucleic Acids Res..

[94] Gebert L.F.R., MacRae I.J. (2019). Regulation of microRNA function in animals. Nat. Rev. Mol. Cell Biol..

[95] Holliday H., Yang J., Dodson E., Nikolic I., Kamili A., Wheatley M., Deng N., Alexandrou S., Davis T.P., Kavallaris M. (2022). miR-99b-5p, miR-380-3p, and miR-485-3p are novel chemosensitizing miRNAs in high-risk neuroblastoma. Mol. Ther..

[96] Liao K., Chen P., Zhang M., Wang J., Hatzihristidis T., Lin X., Yang L., Yao N., Liu C., Hong Y. (2024). Critical roles of the miR-17∼92 family in thymocyte development, leukemogenesis, and autoimmunity. Cell Rep..

[97] Diener C., Hart M., Fecher-Trost C., Knittel J., Rheinheimer S., Meyer M.R., Mayer J., Flockerzi V., Keller A., Meese E. (2023). Outside the limit: questioning the distance restrictions for cooperative miRNA binding sites. Cell. Mol. Biol. Lett..

[98] Chipman L.B., Pasquinelli A.E. (2019). miRNA Targeting: Growing beyond the Seed. Trends Genet..

[99] Zhang K., Zhang X., Cai Z., Zhou J., Cao R., Zhao Y., Chen Z., Wang D., Ruan W., Zhao Q. (2018). A novel class of microRNA-recognition elements that function only within open reading frames. Nat. Struct. Mol. Biol..

[100] Ørom U.A., Nielsen F.C., Lund A.H. (2008). MicroRNA-10a binds the 5'UTR of ribosomal protein mRNAs and enhances their translation. Mol. Cell.

[101] Lehmann S.M., Krüger C., Park B., Derkow K., Rosenberger K., Baumgart J., Trimbuch T., Eom G., Hinz M., Kaul D. (2012). An unconventional role for miRNA: let-7 activates Toll-like receptor 7 and causes neurodegeneration. Nat. Neurosci..

[102] Rezaee D., Saadatpour F., Akbari N., Zoghi A., Najafi S., Beyranvand P., Zamani-Rarani F., Rashidi M.A., Bagheri-Mohammadi S., Bakhtiari M. (2023). The role of microRNAs in the pathophysiology of human central nervous system: A focus on neurodegenerative diseases. Ageing Res. Rev..

[103] Kiel K., Król S.K., Bronisz A., Godlewski J. (2024). MiR-128-3p - a gray eminence of the human central nervous system. Mol. Ther. Nucleic Acids.

[104] Mathiyalagan P., Liang Y., Kim D., Misener S., Thorne T., Kamide C.E., Klyachko E., Losordo D.W., Hajjar R.J., Sahoo S. (2017). Angiogenic Mechanisms of Human CD34(+) Stem Cell Exosomes in the Repair of Ischemic Hindlimb. Circ. Res..

[105] Nammian P., Razban V., Tabei S.M.B., Asadi-Yousefabad S.L. (2020). MicroRNA-126: Dual Role in Angiogenesis Dependent Diseases. Curr. Pharmaceut. Des..

[106] Liu J., Wei E., Wei J., Zhou W., Webster K.A., Zhang B., Li D., Zhang G., Wei Y., Long Y. (2021). MiR-126-HMGB1-HIF-1 Axis Regulates Endothelial Cell Inflammation during Exposure to Hypoxia-Acidosis. Dis. Markers.

[107] Hartmann D., Fiedler J., Sonnenschein K., Just A., Pfanne A., Zimmer K., Remke J., Foinquinos A., Butzlaff M., Schimmel K. (2016). MicroRNA-Based Therapy of GATA2-Deficient Vascular Disease. Circulation.

[108] Chen C., Ling C., Gong J., Li C., Zhang L., Gao S., Li Z., Huang T., Wang H., Guo Y. (2020). Increasing the expression of microRNA-126-5p in the temporal muscle can promote angiogenesis in the chronically ischemic brains of rats subjected to two-vessel occlusion plus encephalo-myo-synangiosis. Aging.

[109] Ma X., Huang Y., He X., Zhang X., Liu Y., Yang Y., Yue P., Liu Y., Gan C., Shu K. (2022). Endothelial Cell-Derived Let-7c-Induced TLR7 Activation on Smooth Muscle Cell Mediate Vascular Wall Remodeling in Moyamoya Disease. Transl. Stroke Res..

[110] Liu J., Chen C., Qin X., Wang J., Zhang B., Jin F. (2024). Plasma-derived exosomes contributes to endothelial-to-mesenchymal transition in Moyamoya disease. Heliyon.

[111] Wu H., Yang L., Chen L.L. (2017). The Diversity of Long Noncoding RNAs and Their Generation. Trends Genet..

[112] Statello L., Guo C.J., Chen L.L., Huarte M. (2021). Gene regulation by long non-coding RNAs and its biological functions. Nat. Rev. Mol. Cell Biol..

[113] Quinn J.J., Chang H.Y. (2016). Unique features of long non-coding RNA biogenesis and function. Nat. Rev. Genet..

[114] Nojima T., Tellier M., Foxwell J., Ribeiro de Almeida C., Tan-Wong S.M., Dhir S., Dujardin G., Dhir A., Murphy S., Proudfoot N.J. (2018). Deregulated Expression of Mammalian lncRNA through Loss of SPT6 Induces R-Loop Formation, Replication Stress, and Cellular Senescence. Mol. Cell.

[115] Schlackow M., Nojima T., Gomes T., Dhir A., Carmo-Fonseca M., Proudfoot N.J. (2017). Distinctive Patterns of Transcription and RNA Processing for Human lincRNAs. Mol. Cell.

[116] Guo C.J., Ma X.K., Xing Y.H., Zheng C.C., Xu Y.F., Shan L., Zhang J., Wang S., Wang Y., Carmichael G.G. (2020). Distinct Processing of lncRNAs Contributes to Non-conserved Functions in Stem Cells. Cell.

[117] Rackham O., Shearwood A.M.J., Mercer T.R., Davies S.M.K., Mattick J.S., Filipovska A. (2011). Long noncoding RNAs are generated from the mitochondrial genome and regulated by nuclear-encoded proteins. RNA.

[118] Mercer T.R., Munro T., Mattick J.S. (2022). The potential of long noncoding RNA therapies. Trends Pharmacol. Sci..

[119] Huang L.A., Lin C., Yang L. (2023). Plumbing mysterious RNAs in "dark genome" for the conquest of human diseases. Mol. Ther..

[120] Mattick J.S., Amaral P.P., Carninci P., Carpenter S., Chang H.Y., Chen L.L., Chen R., Dean C., Dinger M.E., Fitzgerald K.A. (2023). Long non-coding RNAs: definitions, functions, challenges and recommendations. Nat. Rev. Mol. Cell Biol..

[121] Wang K.C., Yang Y.W., Liu B., Sanyal A., Corces-Zimmerman R., Chen Y., Lajoie B.R., Protacio A., Flynn R.A., Gupta R.A. (2011). A long noncoding RNA maintains active chromatin to coordinate homeotic gene expression. Nature.

[122] Mondal T., Subhash S., Vaid R., Enroth S., Uday S., Reinius B., Mitra S., Mohammed A., James A.R., Hoberg E. (2015). MEG3 long noncoding RNA regulates the TGF-β pathway genes through formation of RNA-DNA triplex structures. Nat. Commun..

[123] Kretz M., Siprashvili Z., Chu C., Webster D.E., Zehnder A., Qu K., Lee C.S., Flockhart R.J., Groff A.F., Chow J. (2013). Control of somatic tissue differentiation by the long non-coding RNA TINCR. Nature.

[124] Salmena L., Poliseno L., Tay Y., Kats L., Pandolfi P.P. (2011). A ceRNA hypothesis: the Rosetta Stone of a hidden RNA language?. Cell.

[125] Li X.Z., Xiong Z.C., Zhang S.L., Hao Q.Y., Liu Z.Y., Zhang H.F., Wang J.F., Gao J.W., Liu P.M. (2023). Upregulated LncRNA H19 Sponges MiR-106a-5p and Contributes to Aldosterone-Induced Vascular Calcification via Activating the Runx2-Dependent Pathway. Arterioscler. Thromb. Vasc. Biol..

[126] Gu J., Chen J., Yin Q., Dong M., Zhang Y., Chen M., Chen X., Min J., He X., Tan Y. (2024). lncRNA JPX-Enriched Chromatin Microenvironment Mediates Vascular Smooth Muscle Cell Senescence and Promotes Atherosclerosis. Arterioscler. Thromb. Vasc. Biol..

[127] Zhang L., Tang M., Diao H., Xiong L., Yang X., Xing S. (2023). LncRNA-encoded peptides: unveiling their significance in cardiovascular physiology and pathology-current research insights. Cardiovasc. Res..

[128] Yu J., Wang W., Yang J., Zhang Y., Gong X., Luo H., Cao N., Xu Z., Tian M., Yang P. (2022). LncRNA PSR Regulates Vascular Remodeling Through Encoding a Novel Protein Arteridin. Circ. Res..

[129] Mousavi S.M., Derakhshan M., Baharloii F., Dashti F., Mirazimi S.M.A., Mahjoubin-Tehran M., Hosseindoost S., Goleij P., Rahimian N., Hamblin M.R., Mirzaei H. (2022). Non-coding RNAs and glioblastoma: Insight into their roles in metastasis. Mol. Ther. Oncolytics.

[130] Ma W., Li C.Y., Zhang S.J., Zang C.H., Yang J.W., Wu Z., Wang G.D., Liu J., Liu W., Liu K.P. (2022). Neuroprotective effects of long noncoding RNAs involved in ischemic postconditioning after ischemic stroke. Neural Regen. Res..

[131] Wang W., Gao F., Zhao Z., Wang H., Zhang L., Zhang D., Zhang Y., Lan Q., Wang J., Zhao J. (2017). Integrated Analysis of LncRNA-mRNA Co-Expression Profiles in Patients with Moyamoya Disease. Sci. Rep..

[132] Mamiya T., Kanamori F., Yokoyama K., Ota A., Karnan S., Uda K., Araki Y., Maesawa S., Yoshikawa K., Saito R. (2022). Long noncoding RNA profile of the intracranial artery in patients with moyamoya disease. J. Neurosurg..

[133] Yang M., He X., Huang X., Wang J., He Y., Wei L. (2020). LncRNA MIR4435-2HG-mediated upregulation of TGF-β1 promotes migration and proliferation of nonsmall cell lung cancer cells. Environ. Toxicol..

[134] Hojo M., Hoshimaru M., Miyamoto S., Taki W., Nagata I., Asahi M., Matsuura N., Ishizaki R., Kikuchi H., Hashimoto N. (1998). Role of transforming growth factor-beta1 in the pathogenesis of moyamoya disease. J. Neurosurg..

[135] Yamamoto M., Aoyagi M., Tajima S., Wachi H., Fukai N., Matsushima Y., Yamamoto K. (1997). Increase in elastin gene expression and protein synthesis in arterial smooth muscle cells derived from patients with Moyamoya disease. Stroke.

[136] Weng L., Cao X., Han L., Zhao H., Qiu S., Yan Y., Wang X., Chen X., Zheng W., Xu X. (2017). Association of increased Treg and Th17 with pathogenesis of moyamoya disease. Sci. Rep..

[137] Hartana C.A., Rassadkina Y., Gao C., Martin-Gayo E., Walker B.D., Lichterfeld M., Yu X.G. (2021). Long noncoding RNA MIR4435-2HG enhances metabolic function of myeloid dendritic cells from HIV-1 elite controllers. J. Clin. Invest..

[138] Reho J.J., Guo D.F., Morgan D.A., Rahmouni K. (2021). mTORC1 (Mechanistic Target of Rapamycin Complex 1) Signaling in Endothelial and Smooth Muscle Cells Is Required for Vascular Function. Hypertension.

[139] Kang H.S., Kim J.H., Phi J.H., Kim Y.Y., Kim J.E., Wang K.C., Cho B.K., Kim S.K. (2010). Plasma matrix metalloproteinases, cytokines and angiogenic factors in moyamoya disease. J. Neurol. Neurosurg. Psychiatry.

[140] Xu S., Wei W., Zhang F., Chen T., Dong L., Shi J., Wu X., Zhang T., Li Z., Zhang J. (2022). Transcriptomic Profiling of Intracranial Arteries in Adult Patients With Moyamoya Disease Reveals Novel Insights Into Its Pathogenesis. Front. Mol. Neurosci..

[141] Chen L., Yang Y., Yue R., Peng X., Yu H., Huang X. (2022). Exosomes derived from hypoxia-induced alveolar epithelial cells stimulate interstitial pulmonary fibrosis through a HOTAIRM1-dependent mechanism. Lab. Invest..

[142] Sanger H.L., Klotz G., Riesner D., Gross H.J., Kleinschmidt A.K. (1976). Viroids are single-stranded covalently closed circular RNA molecules existing as highly base-paired rod-like structures. Proc. Natl. Acad. Sci. USA.

[143] Memczak S., Jens M., Elefsinioti A., Torti F., Krueger J., Rybak A., Maier L., Mackowiak S.D., Gregersen L.H., Munschauer M. (2013). Circular RNAs are a large class of animal RNAs with regulatory potency. Nature.

[144] Westholm J.O., Miura P., Olson S., Shenker S., Joseph B., Sanfilippo P., Celniker S.E., Graveley B.R., Lai E.C. (2014). Genome-wide analysis of drosophila circular RNAs reveals their structural and sequence properties and age-dependent neural accumulation. Cell Rep..

[145] Chen L.L. (2016). The biogenesis and emerging roles of circular RNAs. Nat. Rev. Mol. Cell Biol..

[146] Zhang Y., Zhang X.O., Chen T., Xiang J.F., Yin Q.F., Xing Y.H., Zhu S., Yang L., Chen L.L. (2013). Circular intronic long noncoding RNAs. Mol. Cell.

[147] Li Z., Huang C., Bao C., Chen L., Lin M., Wang X., Zhong G., Yu B., Hu W., Dai L. (2015). Exon-intron circular RNAs regulate transcription in the nucleus. Nat. Struct. Mol. Biol..

[148] Feng X.Y., Zhu S.X., Pu K.J., Huang H.J., Chen Y.Q., Wang W.T. (2023). New insight into circRNAs: characterization, strategies, and biomedical applications. Exp. Hematol. Oncol..

[149] Zhang Y., Xue W., Li X., Zhang J., Chen S., Zhang J.L., Yang L., Chen L.L. (2016). The Biogenesis of Nascent Circular RNAs. Cell Rep..

[150] Liang D., Wilusz J.E. (2014). Short intronic repeat sequences facilitate circular RNA production. Genes Dev..

[151] Barrett S.P., Wang P.L., Salzman J. (2015). Circular RNA biogenesis can proceed through an exon-containing lariat precursor. Elife.

[152] Conn S.J., Pillman K.A., Toubia J., Conn V.M., Salmanidis M., Phillips C.A., Roslan S., Schreiber A.W., Gregory P.A., Goodall G.J. (2015). The RNA binding protein quaking regulates formation of circRNAs. Cell.

[153] Liu C.X., Chen L.L. (2022). Circular RNAs: Characterization, cellular roles, and applications. Cell.

[154] Zhong Y., Yang Y., Wang X., Ren B., Wang X., Shan G., Chen L. (2024). Systematic identification and characterization of exon-intron circRNAs. Genome Res..

[155] Okholm T.L.H., Sathe S., Park S.S., Kamstrup A.B., Rasmussen A.M., Shankar A., Chua Z.M., Fristrup N., Nielsen M.M., Vang S. (2020). Transcriptome-wide profiles of circular RNA and RNA-binding protein interactions reveal effects on circular RNA biogenesis and cancer pathway expression. Genome Med..

[156] Du W.W., Yang W., Liu E., Yang Z., Dhaliwal P., Yang B.B. (2016). Foxo3 circular RNA retards cell cycle progression via forming ternary complexes with p21 and CDK2. Nucleic Acids Res..

[157] Zhou Q., Xie D., Wang R., Liu L., Yu Y., Tang X., Hu Y., Cui D. (2022). The emerging landscape of exosomal CircRNAs in solid cancers and hematological malignancies. Biomark. Res..

[158] Gao X., Xia X., Li F., Zhang M., Zhou H., Wu X., Zhong J., Zhao Z., Zhao K., Liu D. (2021). Circular RNA-encoded oncogenic E-cadherin variant promotes glioblastoma tumorigenicity through activation of EGFR-STAT3 signalling. Nat. Cell Biol..

[159] Heo J.I., Ryu J. (2024). Exosomal noncoding RNA: A potential therapy for retinal vascular diseases. Mol. Ther. Nucleic Acids.

[160] Zhou Z., Sun B., Huang S., Zhao L. (2019). Roles of circular RNAs in immune regulation and autoimmune diseases. Cell Death Dis..

[161] Su Q., Lv X. (2020). Revealing new landscape of cardiovascular disease through circular RNA-miRNA-mRNA axis. Genomics.

[162] Mehta S.L., Dempsey R.J., Vemuganti R. (2020). Role of circular RNAs in brain development and CNS diseases. Prog. Neurobiol..

[163] Mao Y.Y., Wang J.Q., Guo X.X., Bi Y., Wang C.X. (2018). Circ-SATB2 upregulates STIM1 expression and regulates vascular smooth muscle cell proliferation and differentiation through miR-939. Biochem. Biophys. Res. Commun..

[164] Liu Y., Huang Y., Zhang X., Ma X., He X., Gan C., Zou X., Wang S., Shu K., Lei T., Zhang H. (2022). CircZXDC Promotes Vascular Smooth Muscle Cell Transdifferentiation via Regulating miRNA-125a-3p/ABCC6 in Moyamoya Disease. Cells.

[165] Nguyen V.N., Motiwala M., Elarjani T., Moore K.A., Miller L.E., Barats M., Goyal N., Elijovich L., Klimo P., Hoit D.A. (2022). Direct, Indirect, and Combined Extracranial-to-Intracranial Bypass for Adult Moyamoya Disease: An Updated Systematic Review and Meta-Analysis. Stroke.

[166] Miyamoto S., Yoshimoto T., Hashimoto N., Okada Y., Tsuji I., Tominaga T., Nakagawara J., Takahashi J.C., JAM Trial Investigators (2014). Effects of extracranial-intracranial bypass for patients with hemorrhagic moyamoya disease: results of the Japan Adult Moyamoya Trial. Stroke.

[167] Kuroda S., Fujimura M., Takahashi J., Kataoka H., Ogasawara K., Iwama T., Tominaga T., Miyamoto S., Research Committee on Moyamoya Disease Spontaneous Occlusion of Circle of Willis of the Ministry of Health, Labor, and Welfare, Japan (2022). Diagnostic Criteria for Moyamoya Disease - 2021 Revised Version. Neurol. Med.-Chir..

[168] Sun H., Li Y., Xiao A., Li W., Xia C., You C., Ma L., Liu Y., Xia C. (2023). Nomogram to Predict Good Collateral Formation After the STA-MCA Bypass Surgery in Adult Patients With Moyamoya Disease. Stroke.

[169] Ge P., Ye X., Liu X., Deng X., Wang J., Wang R., Zhang Y., Zhang D., Zhang Q., Zhao J. (2019). Angiographic Outcomes of Direct and Combined Bypass Surgery in Moyamoya Disease. Front. Neurol..

[170] Zhang J., Li S., Fujimura M., Lau T.Y., Wu X., Hu M., Zheng H., Xu H., Zhao W., Li X., Chen J. (2019). Hemodynamic analysis of the recipient parasylvian cortical arteries for predicting postoperative hyperperfusion during STA-MCA bypass in adult patients with moyamoya disease. J. Neurosurg..

[171] Tao T., Zhu W., Yu J., Li X., Wei W., Hu M., Luo M., Wan G., Li P., Chen J., Zhang J. (2024). Intraoperative evaluation of local cerebral hemodynamic change by laser speckle contrast imaging for predicting postoperative cerebral hyperperfusion during STA-MCA bypass in adult patients with moyamoya disease. J. Cerebr. Blood Flow Metabol..

[172] Schübeler D. (2015). Function and information content of DNA methylation. Nature.

[173] Wu X., Zhang Y. (2017). TET-mediated active DNA demethylation: mechanism, function and beyond. Nat. Rev. Genet..

[174] Morris-Blanco K.C., Kim T., Lopez M.S., Bertogliat M.J., Chelluboina B., Vemuganti R. (2019). Induction of DNA Hydroxymethylation Protects the Brain After Stroke. Stroke.

[175] Lo Y.M.D., Han D.S.C., Jiang P., Chiu R.W.K. (2021). Epigenetics, fragmentomics, and topology of cell-free DNA in liquid biopsies. Science.

[176] Lo Y.M., Corbetta N., Chamberlain P.F., Rai V., Sargent I.L., Redman C.W., Wainscoat J.S. (1997). Presence of fetal DNA in maternal plasma and serum. Lancet.

[177] Wan J.C.M., Massie C., Garcia-Corbacho J., Mouliere F., Brenton J.D., Caldas C., Pacey S., Baird R., Rosenfeld N. (2017). Liquid biopsies come of age: towards implementation of circulating tumour DNA. Nat. Rev. Cancer.

[178] Luo H., Wei W., Ye Z., Zheng J., Xu R.H. (2021). Liquid Biopsy of Methylation Biomarkers in Cell-Free DNA. Trends Mol. Med..

[179] Lo Y.M., Tein M.S., Pang C.C., Yeung C.K., Tong K.L., Hjelm N.M. (1998). Presence of donor-specific DNA in plasma of kidney and liver-transplant recipients. Lancet.

[180] Agbor-Enoh S., Shah P., Tunc I., Hsu S., Russell S., Feller E., Shah K., Rodrigo M.E., Najjar S.S., Kong H. (2021). Cell-Free DNA to Detect Heart Allograft Acute Rejection. Circulation.

[181] De Vlaminck I., Valantine H.A., Snyder T.M., Strehl C., Cohen G., Luikart H., Neff N.F., Okamoto J., Bernstein D., Weisshaar D. (2014). Circulating cell-free DNA enables noninvasive diagnosis of heart transplant rejection. Sci. Transl. Med..

[182] Schütz E., Fischer A., Beck J., Harden M., Koch M., Wuensch T., Stockmann M., Nashan B., Kollmar O., Matthaei J. (2017). Graft-derived cell-free DNA, a noninvasive early rejection and graft damage marker in liver transplantation: A prospective, observational, multicenter cohort study. PLoS Med..

[183] Cheng A.P., Cheng M.P., Loy C.J., Lenz J.S., Chen K., Smalling S., Burnham P., Timblin K.M., Orejas J.L., Silverman E. (2022). Cell-free DNA profiling informs all major complications of hematopoietic cell transplantation. Proc. Natl. Acad. Sci. USA.

[184] Zhang S., Zhan L., Li X., Yang Z., Luo Y., Zhao H. (2021). Preclinical and clinical progress for HDAC as a putative target for epigenetic remodeling and functionality of immune cells. Int. J. Biol. Sci..

[185] Zhang Y., Zhang G., Wang Y., Ye L., Peng L., Shi R., Guo S., He J., Yang H., Dai Q. (2024). Current treatment strategies targeting histone deacetylase inhibitors in acute lymphocytic leukemia: a systematic review. Front. Oncol..

